# Out-of-Pocket Expenditure (OOPE) on Selected Surgeries in the Obstetrics and Gynaecology Department incurred by Ayushman Bharat Pradhan Mantri Jan Arogya  Yojana (AB-PMJAY), Private Health Insurance and Uninsured Patients in a Tertiary Care Teaching Hospital in Karnataka state of India.

**DOI:** 10.12688/f1000research.157203.2

**Published:** 2025-01-28

**Authors:** Sagarika Kamath, Siddhartha Sankar Acharya, Helmut Brand, Prajwal Salins, Reena Verma, Dr. Kumar Sumit, Dr. Vidya Prabhu, rajesh kamath

**Affiliations:** 1 Department of International Health, Care and Public Health Research Institute – CAPHRI, Faculty of Health, Medicine and Life Sciences,, Maastricht University, Maastricht, The Netherlands; 2 Prasanna School of Public Health, Manipal Academy of Higher Education, Manipal, India; 3 Prasanna School of Public Health, Manipal Academy of Higher Education, Manipal, India; 4 Assistant Professor, Department of Health Information Management, Manipal college of health professions, Manipal Academy of Higher Education, Manipal, India; 5 Associate Professor, Welcomgroup Graduate School of Hotel Administration, Manipal Academy of Higher Education, Manipal, India; 6 Associate Professor, Department of Global public health policy and Governance, Prasanna School of Public Health, Manipal Academy of Higher Education, Manipal, India; 7 Assistant Professor – senior scale, Department of Healthcare and Hospital Management, Prasanna School of Public Health, Manipal Academy of Higher Education, Manipal, India

**Keywords:** Out-of Pocket Expenditure, AB-PMJAY, Ayushman Bharat, Health Insurance, Maternal Mortality Rate, Maternal Health, Obstetrics and Gynaecology, Universal Health Coverage

## Abstract

**Introduction:**

OOPE for healthcare services is a major concern within the Indian healthcare system. 30% of the population remains uninsured despite increasing health insurance coverage. For obstetrics and gynaecology (OBG) patients financial obstacles like OOPE can delay access to health care, evaluating spending patterns can inform policies to enhance accessibility, affordability and equitable health.

**Methodology:**

A retrospective study was conducted at a tertiary care teaching hospital in Karnataka state of India to analyze OOPE for 905 OBG patients who underwent Cesarean Section(C-Section), Laparoscopic Hysterectomy, Laparoscopic Cystectomy, Laparoscopic Myomectomy and Laparoscopic Assisted Vaginal Hysterectomy (LAVH). These were the top five most performed obstetrics and gynecology surgeries in the tertiary care teaching hospital between January 2023 and July 2023. Data was collected across AB-PMJAY, private health insurance, uninsured patients and analyzed using descriptive statistics (mean, median) and the Shapiro-Wilk test for data normality.

**Results:**

The study analyzed OOPE across 905 OBG patients. Findings show AB-PMJAY provided full coverage with zero OOPE for all surgeries. Though private health insurance reduced OOPE compared to uninsured patients under private health insurance still faced significant financial burden. Variations existed in minimum and maximum OOPE and percentage of OOPE across different private insurances. All the uninsured patients incurred 100% OOPE, with a median OOPE of Rs. 33,257 (405.67 USD) to Rs. 57,053 (695.76 USD) and a mean OOPE of Rs. 39,848 (485.95 USD) to Rs. 60,687 (740 USD) across the surgeries.

**Discussion:**

Findings of the study show that AB-PMJAY is highly effective in reducing OOPE and enhancing financial risk protection for OBG patients. Patients covered by private health insurance incurred less OOPE, yet the burden remained considerable. High OOPE rates for uninsured patients (100%) and private health insurance (38.15%) compared to 0% OOPE in AB-PMJAY emphasizes the need for the continued expansion of AB-PMJAY.

## Introduction

Out-of-pocket expenditure (OOPE) associated with healthcare services have become a significant global concern, including in India. The healthcare sector in India has experienced a significant rise in private-sector participation in recent years. According to National Sample Survey Organization (NSSO) in the fiscal year 2017-18, it was found that private healthcare facilities constituted 55.3% of total hospitalizations. ^1^

The private healthcare sector in India exhibits high OOPE, which leads to a diminished level of financial protection for patients. Although there has been a decrease in the proportion of OOPE in relation to total healthcare expenditure (THE), from 62.6% (Rs. 3,02,520 crore or 39.08 Billion USD) in 2014-15 to 47.1% (Rs. 2,80,923 crore or 34.25 Billion USD) in 2019-20, the burden of OOPE still remains significant. ^1^ In Karnataka, THE for the fiscal year 2019-20 amounted to Rs. 35,761crores (4.36 Billion USD) and OOPE accounted for 31.8%, which is equivalent to Rs. 11,368 crores (1.38 Billion USD). ^2^

The aggregate size of the health insurance market, as measured by the total premium collected, experienced a significant increase from Rs. 44,873 crores (5.47 Billion USD) in the fiscal year 2018-19 to Rs. 73,052 crores (8.90 Billion USD) in the fiscal year 2021-22. ^3^ The Private Health Insurance market has experienced significant growth in terms of premium collection, increasing from Rs. 10,655 crore (1.29 Billion USD) in the fiscal year 2018-19 to Rs. 20,107 crore (2.45 Billion USD) in the fiscal year 2021-22. ^3^ Despite the considerable growth, private health insurance continues to offer limited coverage, reaching only 9% of the total population. ^4^ 70% of the Indian population is estimated to be covered under public and private health insurance as shown in Table 1. ^4– 7^ 30% of the population (40 crores or 0.4 Billion) does not have any type of health insurance coverage. ^8^

**Table 1.** The number of eligible or covered individuals and families, categorized by the type of health insurance scheme.

Insurance Scheme	Individuals Eligible or Covered (cr.)	Percentage of Population Eligible	Families Eligible or Covered (cr.)
**Government Subsidized Schemes**	69 (690 Million)	51%	15.3(153 Million)
AB-PMJAY (w/o State Extension Schemes)	49 (490 Million)	36%	10.9 (109 Million)
AB-PMJAY State Extension Schemes	20 (200 Million)	15%	4.4 (440 Million)
**Social Health Insurance Schemes**	14 (140 Million)	10%	3.6 (36 Million)
Employees' State Insurance Scheme (ESIS)	13.6 (136 Million)	10%	3.5 (35 Million)
Central Government Health Scheme	0.4 (40 million)	0.3%	0.13 (1.3 Million)
**Private Voluntary Health Insurance (PHI)**	11.5 (115 Million)	9%	2.6 (26 Million)
**Total Eligible or Covered (**assuming no overlap)	94.5 (945 Million)	70%	21.5 (215 Million)
**Total Population/Families**	135 (1350 Million)		30 (300 Million)
**Uncovered Population/Families**	40.5 (405 Million)	30%	8.5 (85 Million)

AB-PMJAY was launched in 2018 by the government of India as a direct response to the demand for comprehensive healthcare services serving the vulnerable population of the country. AB-PMJAY is a government-funded healthcare program aimed at providing health insurance coverage to more than 50 crore (500 Million) individuals across India. It is recognized as one of the largest healthcare initiatives of its kind. As of March 19, 2023, e.g. the state of Karnataka has generated more than 1.36 crore or 13.6 Million AB-PMJAY cards. A total of 35,88,000 or 3.58 Million beneficiaries have availed free healthcare services at an expenditure of Rs.2,982 crore. ^9^ One of the vital aspects of AB-PMJAY is the provision of free maternity care, aligning with the objectives of the National Health Policy (NHP) 2017. The primary objective of this policy is to reduce India’s maternal mortality rate (MMR) to below 100 per 100,000 live births by 2020. India has achieved this objective by reducing the MMR to 97 per 100,000 live births in 2020. ^10^

The United Nations has established Target 3.1 of the Sustainable Development Goals (SDGs) to address the worldwide emphasis on maternal health. The current aim of the SDGs is to achieve a worldwide decrease in MMR to less than 70 per 100,000 live births by the year 2030. To achieve Target 3.1 of the SDGs, it is important to improve interventions in areas where MMR remains high, ensuring adequate access to high-quality maternal healthcare services throughout India. ^10^ There also exist challenges notably evident in the MMR observed in some specific Indian states. Meghalaya demonstrates a significantly high MMR of 197 Assam following closely at 195, Madhya Pradesh at 173, Uttar Pradesh at 167, Chhattisgarh at 137, Odisha at 119, Bihar at 118. ^11, 12^

The disparities in MMR across different Indian states emphasize the urgent need for focused interventions aimed at mitigating disparities in MMR. The disparity in MMR can be attributed to several factors, such as economic status, education, restricted availability of healthcare services, lack of awareness regarding obstetrics and gynaecology that impact individuals’ healthcare-seeking tendencies. ^13, 14^ States and Union Territories that have a significant proportion of institutional deliveries, such as Lakshadweep (100%), Tamil Nadu (99.99%), Puducherry (99.98%), Kerala (99.82%) and Karnataka (99.74%) demonstrate a lower MMR. On the other hand, states such as Meghalaya (57.79%), Bihar (81.27%), Uttar Pradesh (85.63%) and Assam (89.88%), which exhibit lower proportions of institutional deliveries, are associated with elevated levels of MMR. ^15^ The correlation between institutional delivery and MMR highlights the importance of institutional deliveries in mitigating MMR.

Laparoscopic Hysterectomy, Laparoscopic Myomectomy, Laparoscopic Cystectomy and LAVH are minimally invasive surgical procedures widely performed for the management of various gynaecological conditions. Laparoscopic Myomectomy is indicated for the removal of uterine fibroids, benign tumors that can cause symptoms such as abnormal uterine bleeding, pelvic pain and infertility. Laparoscopic Cystectomy is performed to remove ovarian cysts or masses, which can be associated with pelvic pain and irregular menstrual cycles. LAVH and Laparoscopic hysterectomy are treatment options for conditions such as uterine prolapse, menorrhagia, uterine fibroids and endometriosis where the uterus is removed through a combination of laparoscopic and vaginal approaches.

In the year 2016, the total number of hysterectomy in India was estimated to be approximately 10 million among women aged 30-49 years. ^16^ A study in low-income households in Gujarat, India, found nearly two-thirds of rural women who underwent hysterectomies sought care in private hospitals, resulting in a high OOPE. ^17^ A study in Nigeria found that the absence of an effective health insurance scheme led to an increase in OOPE to Laparsocopic Myomectomy, Laparoscopic Cystectomy and LAVH. ^18^

A positive correlation between possession of health insurance and increased utilization of obstetrics and gynaecology healthcare services has been found. ^19– 22^ The correlation highlights the importance of health insurance in improving the delivery of obstetrics and gynaecology healthcare services. The increase in women’s enrolment in health insurance is recognized as a critical approach to improving the utilization of obstetrics and gynaecology healthcare services. ^12, 20^ Obstetrics and gynaecology patients patients from low-income households and marginalized communities encounter financial hardships as a result of OOPE. A study conducted in two villages near Delhi in north India, found that 43% of women who were married to laborers or unemployed individuals gave birth at home. ^23^ A significant association between the place of childbirth and various economic indicators such as the occupation of the spouse, monthly income and socio-economic status have been confirmed. ^23^

Obstetrics and gynaecology patients patients from low-income households face financial obstacles that delay their timely access to healthcare facilities, thereby contributing to unfavourable outcomes and influencing MMR. ^24, 25^ Understanding and addressing OOPE is highly significant, as they have a substantial influence on MMR, particularly for obstetrics and gynaecology patients patients from socioeconomically disadvantaged groups. OOPE has the potential to result in financial burden on individuals, leading to impoverishment and hindering timely access to healthcare services. Financial limitations and OOPE may also lead to a change in maternal health behaviours such as compromising on dietary intake or skipping necessary prenatal examinations in order to save money. These behaviours have the potential to contribute to health complications which can result in negative outcomes for the mother and can also potentially contribute to MMR.

High OOPE can lead to catastrophic health expenditure, defined as healthcare costs exceeding a significant proportion of household income, forcing households to forgo essential needs, incur debt, or fall into poverty. ^26^ In India, approximately 30% of the population remains uninsured and must bear the full financial burden of healthcare services. ^8^ For low-income households, catastrophic expenditure can delay timely access to care, exacerbate health inequities, and contribute to impoverishment. ^22, 24, 25^ Women in rural Gujarat have reported high OOPE for hysterectomies, primarily due to the absence of financial risk protection. ^17^ By mitigating OOPE, government schemes like AB-PMJAY play a critical role in preventing catastrophic health expenditure and alleviating poverty for vulnerable population. ^27^

While AB-PMJAY and private health insurance offer potential remedies, their impact on OOPE incurred for obstetrics and gynaecology patient needs further investigation. This study aims to conduct a focused investigation across that directly compare obstetrics and gynaecology patients patients’ OOPE during Cesarian-Section (C-Section), Laparoscopic Hysterectomy, Laparoscopic Assisted Vaginal Hysterectomy (LAVH), Laparoscopic Myomectomy and Laparoscopic Cystectomy conducted within the Obstetrics and Gynaecology department of a tertiary care teaching hospital in coastal Karnataka, India. C-Section, laparoscopic hysterectomy, laparoscopic cystectomy, laparoscopic myomectomy and LAVH were the top five most performed obstetrics and gynecology surgeries in the tertiary care teaching hospital between January 2023 and July 2023 and scrutinizing spending patterns associated with these five surgeries can provide precise insights for refining policies to enhance the accessibility of obstetrics and gynaecology patients and to mitigate the financial burden.

## Methods

### Study setting

A retrospective study was conducted over a period of three months starting from December 2023 to February 2024 in a tertiary care teaching hospital in Karnataka state of India. The hospital is a 2000 bedded hospital. Every day, the hospital serves about 200 inpatients and 2,500 outpatients. The hospital is a National Accreditation Board for Hospitals and Healthcare Providers (NABH) accredited hospital. Patients who have had a C-section, Laparoscopic Hysterectomy, Laparoscopic Myomectomy, Laparoscopic Cystectomies and Laparoscopic Assisted Vaginal Hysterectomy (LAVH) were considered for the study. These five surgeries were considered for the study as these five surgeries represented the highest number of obstetrics and gynaecology surgeries in the tertiary care teaching hospital with a total of 783 C-Section cases, 44 cases of Laparoscopic Hysterectomy, 30 cases of Laparoscopic Cystectomy, 18 cases of Laparoscopic Myomectomy, and 30 cases of LAVH between January 2023 and July 2023.

### Study design

Retrospective single centric study.

### Departments involved

▪ Medical record department
▪ IT department
▪ Finance department

### Inclusion criteria

▪ Patients who have undergone C-Section, Laparoscopic Hysterectomy, Laparoscopic Assisted Vaginal Hysterectomy (LAVH), Laparoscopic Myomectomy and Laparoscopic Cystectomy.
▪ Patients who are covered by AB-PMJAY, private health insurance and uninsured patients.
▪ Surgeries performed between January 2023 to July 2023.

### Exclusion criteria

▪ Patients covered by health insurance other than AB-PMJAY and private health insurance.
▪ Multiple pregnancies
▪ Patients who have undergone other types of surgeries within the same department as well as different departments.
▪ Maternal death

### Sample size

Data of 905 obstetrics and gynaecology patients were considered for this study.

### Tools used

A validated proforma was used. The validated proforma is available in the data repository mentioned in the data availability statement below.

### Statistical method

Descriptive statistics such as mean, median and the Shapiro-Wilk test to check the normality of the data were used to analyse and compare the levels of variability in OOPE among different patient category such as AB-PMJAY, private health insurance and uninsured patients.

***In the context of the study, OOPE** is defined as* the portion of the hospitalization bill paid directly by the patient or their family, which is not covered by any insurance scheme or third-party payer. This includes costs related to hospital admission, medications, diagnostic tests, surgical procedures, consumables, and any additional charges incurred during hospitalization or treatment.

### Ethical consideration

Institutional Ethics Committee (IEC) approval was granted from the Kasturba Medical College and Kasturba Hospital Institutional Ethics Committee – 2: DHR registration no. EC/NEW/INST/2021/1707 (IEC2:562/2023) on 19-1-24. The first application was made to the IEC on 12/09/2023. The IEC asked for Modifications (# IEC form: Risk is minimal. # Permission from HOD OBG required. # Inter institute research form to be filled) on 01/11/2023. The first IEC Approval was secured on 21/11/2023. An application for an amendment (addition of co-guide) was made on: 28/12/2023. The 2nd IEC approval date is 19/01/2024. The patient data was made anonymous and treated with strict confidentiality for the study. As the study is a retrospective study and did not involve any new treatments or interventions, informed consent was not applicable. Consent was waived by the ethics committee.

## Results

The study included 905 obstetrics and gynaecology patients, who underwent C-Section, Laparoscopic Hysterectomy, Laparoscopic Cystectomy, Laparoscopic Myomectomy and LAVH and uninsured or were covered by AB-PMJAY or private health insurance from January 2023 to July 2023.

**Figure 1.**
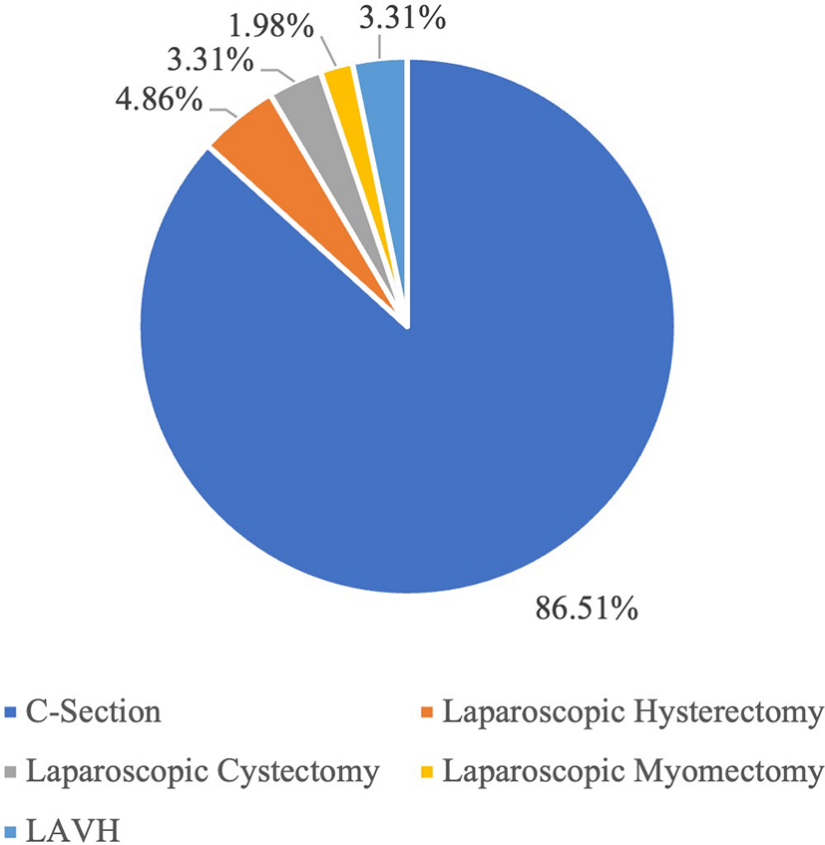
The figure shows the proportion of each category of the surgeries within a total of 905 surgeries. Percentage-wise distribution of surgeries across various categories. ^38^

Table 2 presents the comprehensive distribution of 905 cases across the five surgeries. The table outlines the specific breakdown, comprising 783 C-Section cases, 44 cases of Laparoscopic Hysterectomy, 30 cases of Laparoscopic Cystectomy, 18 cases of Laparoscopic Myomectomy and 30 cases of Laparoscopic Assisted Vaginal Hysterectomy (LAVH). Figure 1 visually depicts this distribution, presenting the representation of each surgical category within the total of 905 surgeries. The corresponding percentages for each category are as follows: 86.51% for C-Section, 4.86% for Laparoscopic Hysterectomy, 3.31% for Laparoscopic Cystectomy, 1.98% for Laparoscopic Myomectomy and 3.31% for LAVH.

**Table 2.** Distribution of surgeries across various categories.

Surgeries	No. of cases
C-Section	783
Laparoscopic Hysterectomy	44
Laparoscopic Cystectomy	30
Laparoscopic Myomectomy	18
LAVH	30
**Total**	**905**

Table 3 presents the number of cases, median and mean billing amount of the selected surgeries. Under AB-PMJAY, total number of cases of C-Section, Laparoscopic Hysterectomy, Laparoscopic Cystectomy, Laparoscopic Myomectomy and LAVH were 51, 11, 7, 6, and 11 respectively. Under private health insurance, total number of cases of C-Section, Laparoscopic Hysterectomy, Laparoscopic Cystectomy, Laparoscopic Myomectomy and LAVH were 201, 11, 11, 3, and 6 respectively. For uninsured patients, total number of cases of C-Section, Laparoscopic Hysterectomy, Laparoscopic Cystectomy, Laparoscopic Myomectomy and LAVH were 531, 22, 12, 9 and 13 respectively. Under AB-PMJAY, the median billing amount for C-Section, Laparoscopic Hysterectomy, Laparoscopic Cystectomy and LAVH was Rs. 9000 (109.75 USD), Rs. 20,000 (244 USD) and Rs. 11,500 (140.24 USD), while the mean billing amount for Laparoscopic Myomectomy was Rs. 15,000 (183 USD). Under private health insurance, the median billing amount for C-Section was Rs. 50,433 (615 USD). The mean billing amount for Laparoscopic Hysterectomy, Laparoscopic Cystectomy, Laparoscopic Myomectomy and LAVH was Rs. 84,994 (1036.5 USD), Rs. 50,991 (623 USD), Rs. 29,604 (361 USD) and Rs. 58,501(713.42 USD) respectively. For uninsured patients, the median billing amount for C-Section, Laparoscopic Hysterectomy and Laparoscopic Cystectomy was Rs. 33,257 (406 USD), Rs. 57,053 (696 USD) and Rs. 35,891 (438 USD) respectively. The mean billing amount for Laparoscopic Myomectomy and LAVH was Rs. 39,848 (485 USD) and Rs. 53,151 (648.25 USD) respectively. OOPE as average percentage of billing amount was zero for AB-PMJAY across the selected surgeries. For private health insurance it ranged from 15.05% to 38.15% across the selected surgeries. For uninsured patients it was 100% across the selected surgeries. For, AB-PMJAY, median and mean OOPE was zero across the selected surgeries. For private health insurance it ranged from Rs. 5,435 (66.28 USD) to Rs. 35,891(438 USD) across the surgeries. For uninsured patients it ranged from Rs. 33,275 (406 USD) to Rs. 57,053 (696 USD).

**Table 3.** Type of Surgery, Number of Cases, Median and Mean Billing Amount, OOPE as average Percentage (%) of Billing Amount, Median and Mean OOPE. ^38^

Patient Category	Type of Surgery	No. of cases	Total Percentage of Cases (%)	Median Billing Amount/Mean Billing Amount (INR)	OOPE as average Percentage (%) of Billing Amount	Median OOPE/Mean OOPE (INR)
**AB-PMJAY **	C-Section	51	5.64	9,000 * ($109.75)	0	0
	Laparoscopic Hysterectomy	11	1.22	20,000 * ($244)	0	0
	Laparoscopic Cystectomy	7	0.77	15,000 * ($183)	0	0
	Laparoscopic Myomectomy	6	0.66	15,000 ** ($183)	0	0
	LAVH	11	1.22	11,500 * ($140.24)	0	0
	**Total**	**86**	**9.5**			
**Private Health Insurance**	C-Section	201	22.21	50,433 * ($615)	38.15	17,426 *** ($212.51)
	Laparoscopic Hysterectomy	11	1.22	84,994 ** ($1036.5)	19.23	7,600 *** ($93)
	Laparoscopic Cystectomy	11	1.22	50,991 ** ($623)	23.64	35,891 *** ($438)
	Laparoscopic Myomectomy	3	0.33	29,604 ** ($361)	18.58	5,435 **** ($66.28)
	LAVH	6	0.66	58,501 ** ($713.42)	15.05	7,230 **** ($88.17)
	**Total**	**232**	**25.64**			
**Uninsured Patients**	C-Section	531	58.67	33,257 * ($406)	100	33,275 *** ($406)
	Laparoscopic Hysterectomy	22	2.43	57,053 *($696)	100	57,053 *** ($696)
	Laparoscopic Cystectomy	12	1.33	35,891 * ($438)	100	35,891 *** ($438)
	Laparoscopic Myomectomy	9	0.99	39,848 ** ($485)	100	39,848 ****($486)
	LAVH	13	1.44	53,151 ** ($648.25)	100	53,151 ****($648.18)
	**Total**	**587**	**64.86%**			
**Grand Total of All Surgeries**		**905**				

* Median billing was considered to measure the central tendency for billing amount where p-value in Shapiro-Wilk test was <0.05, indicating significant deviation from a normal distribution. (e.g. C-Section under AB-PMJAY).

** Mean billing was considered where p-value was >0.05, indicating normal distribution (e.g. Laparoscopic Hysterectomy under Private Health Insurance).

*** Median OOPE was considered to measure the central tendency for billing amount where p-value in Shapiro-Wilk test was <0.05, indicating significant deviation from a normal distribution. (e.g. C-Section under Private Health Insurance).

**** Mean OOPE was considered where p-value was >0.05, indicating normal distribution (e.g. Laparoscopic Myomectomy under Private Health Insurance).

### Total billing of the selected surgeries

**Figure 2.**
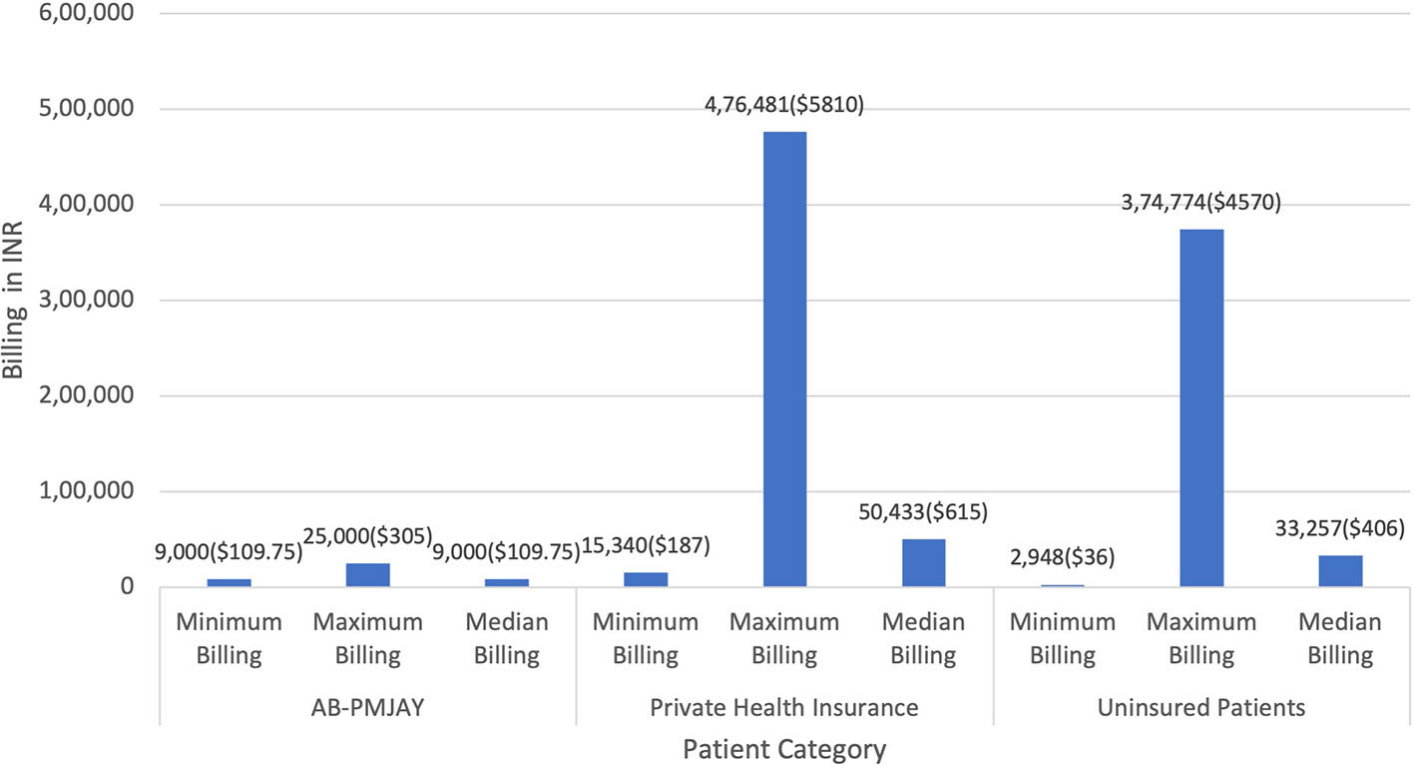
The figure compares minimum, maximum, and median billing amounts for C-sections across AB-PMJAY, private insurance, and uninsured patients. C-Section Billing: Minimum, Maximum and Median. ^38^

Figure 2 presents the minimum, maximum and median billing amounts for C-section across AB-PMJAY, private health insurance and uninsured patients. AB-PMJAY billing ranged from a minimum of Rs.9,000 (109.75 USD), to a maximum of Rs.25,000 (305 USD), with a median of Rs.9,000 (109.75 USD),. Private health insurance billing had a larger range, from a minimum of Rs.15,340 (187.07 USD) to a maximum of Rs.4,76,481 (5811 USD), with a median billing of Rs.50433 (615.03 USD). Uninsured patients fell in the middle, with a minimum billing of Rs.2,948 (36 USD), maximum of Rs.3,74,774 (4570.41 USD), and median of Rs.33,257 (406 USD). Here median was used as the main statistic for central tendency since the Shapiro-Wilk test yielded a p-value less than 0.05, indicating significant deviation from a normal distribution.

The findings show that billing amounts can vary among AB-PMJAY, private health insurance, and uninsured patients. AB-PMJAY beneficiaries are typically assigned to general wards, while private health insurance offers a limited selection of beds. The choice of bed type affects the total billing amount, nursing charges, and physician consultation fees. While uninsured patients can choose the bed category as per their requirements.

**Figure 3.**
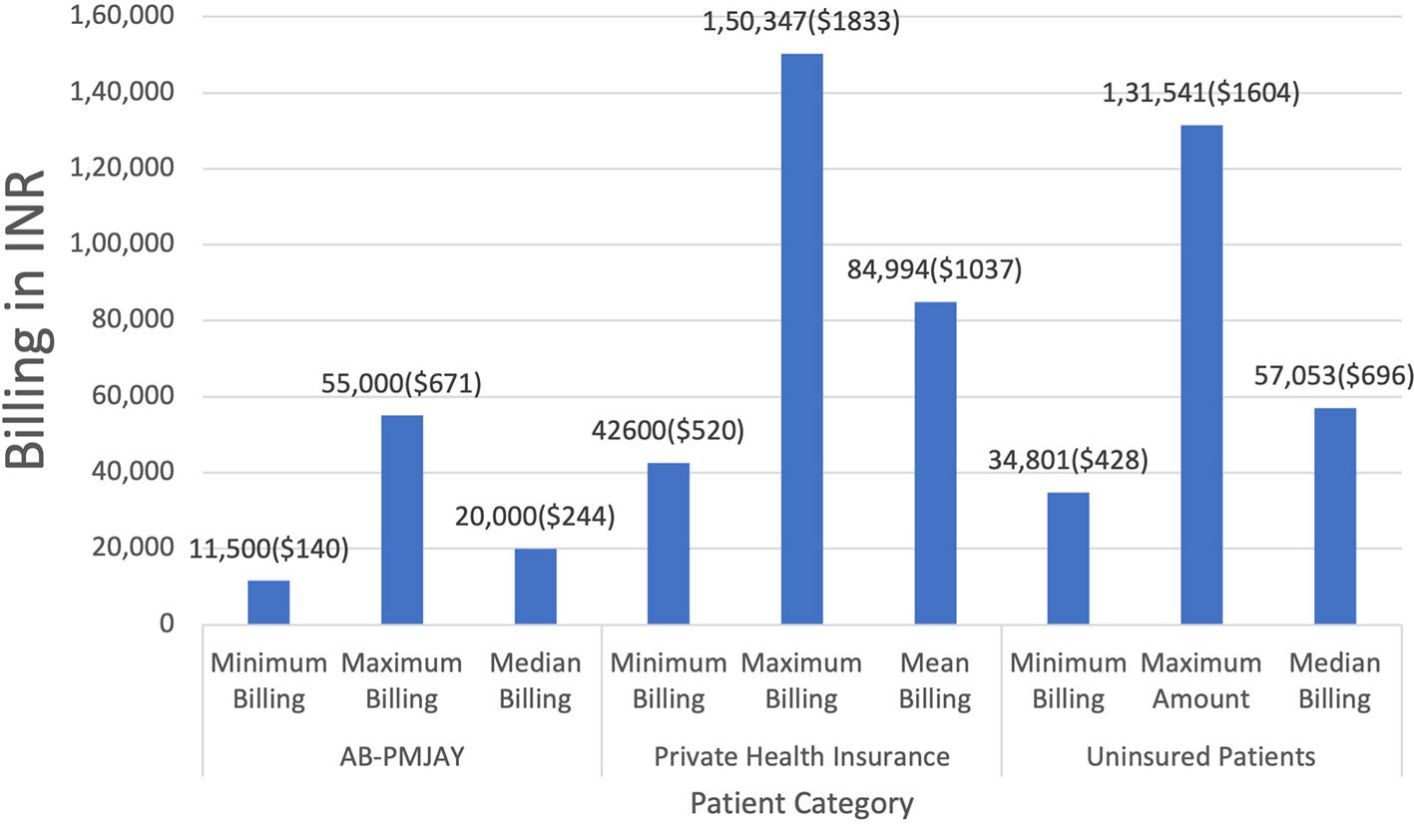
The figure shows the minimum, maximum, median, and mean billing amounts for Laparoscopic Hysterectomy across AB-PMJAY, private insurance, and uninsured patients. Laparoscopic Hysterectomy billing: Minimum, Maximum, Median and Mean. ^38^

Figure 3 presents the minimum, maximum, median and mean billing amounts for Laparoscopic Hysterectomy across AB-PMJAY, private health insurance and uninsured patients. AB-PMJAY, ranged from a minimum of Rs. 11,500 (140 USD) to a maximum of Rs.55,000 (671 USD), with a median of Rs.20,000 (244 USD). Private insurance ranged from a minimum of Rs.42,600 (520 USD) to a maximum of Rs.1,50,347 (1833 USD) with a mean of Rs.84,994 (1037 USD). Uninsured patients ranged with a minimum billing of Rs.34,801(424 USD) to a maximum of Rs.1,31,541(1604 USD) and median of Rs.57,053 (696 USD). Here median was used as the main statistic to measure the central tendency of AB-PMJAY and uninsured patients as the Shapiro-Wilk test yielded a p-value less than.005, indicating significantly deviation from a normal distribution. While for private health insurance, mean was used as the data was normally distributed (p>0.05).

**Figure 4.**
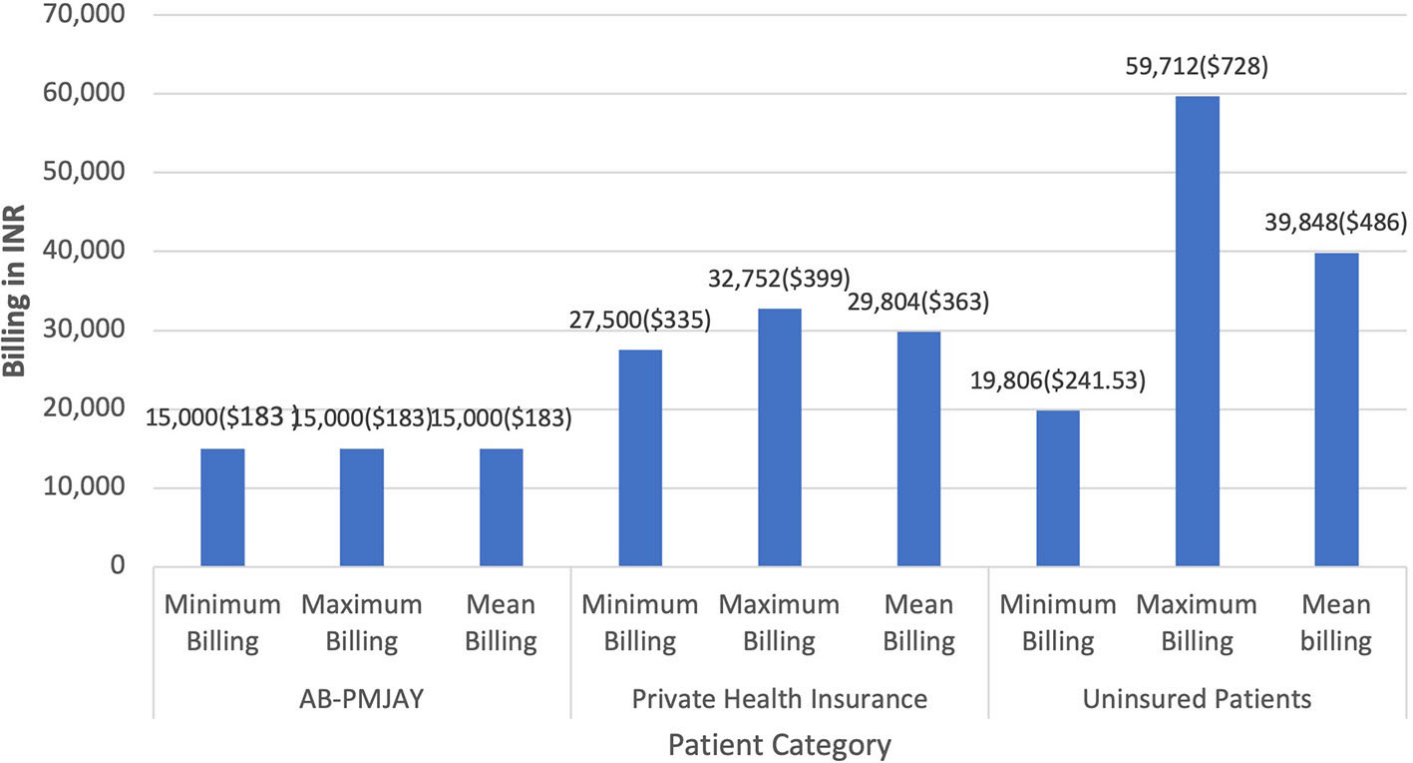
The figure shows the minimum, maximum, and mean billing amounts for Laparoscopic Myomectomy across AB-PMJAY, private insurance, and uninsured patients. Laparoscopic Myomectomy billing: Minimum, Maximum and Mean. ^38^

Figure 4 presents the minimum, maximum and mean of billing amount for Laparoscopic Myomectomy across AB-PMJAY, private health insurance and uninsured patients. AB-PMJAY had a minimum and maximum billing of Rs. 15,000 (183 USD) with a mean of Rs.15,000 (183 USD). Private insurance ranged from a minimum of Rs.27,500 (335 USD) to a maximum of Rs.32,752 (399 USD) with a mean of Rs.29,804 (363 USD). Uninsured patients ranged with a minimum billing of Rs. 19,806 (241.53 USD) to a maximum of Rs. 59,712 (728 USD) and mean of Rs.39,848 (486 USD). Here mean was used as the main statistic to measure the central tendency as the Shapiro-Wilk test yielded a p-value of more than 0.05, indicating normal distribution.

**Figure 5.**
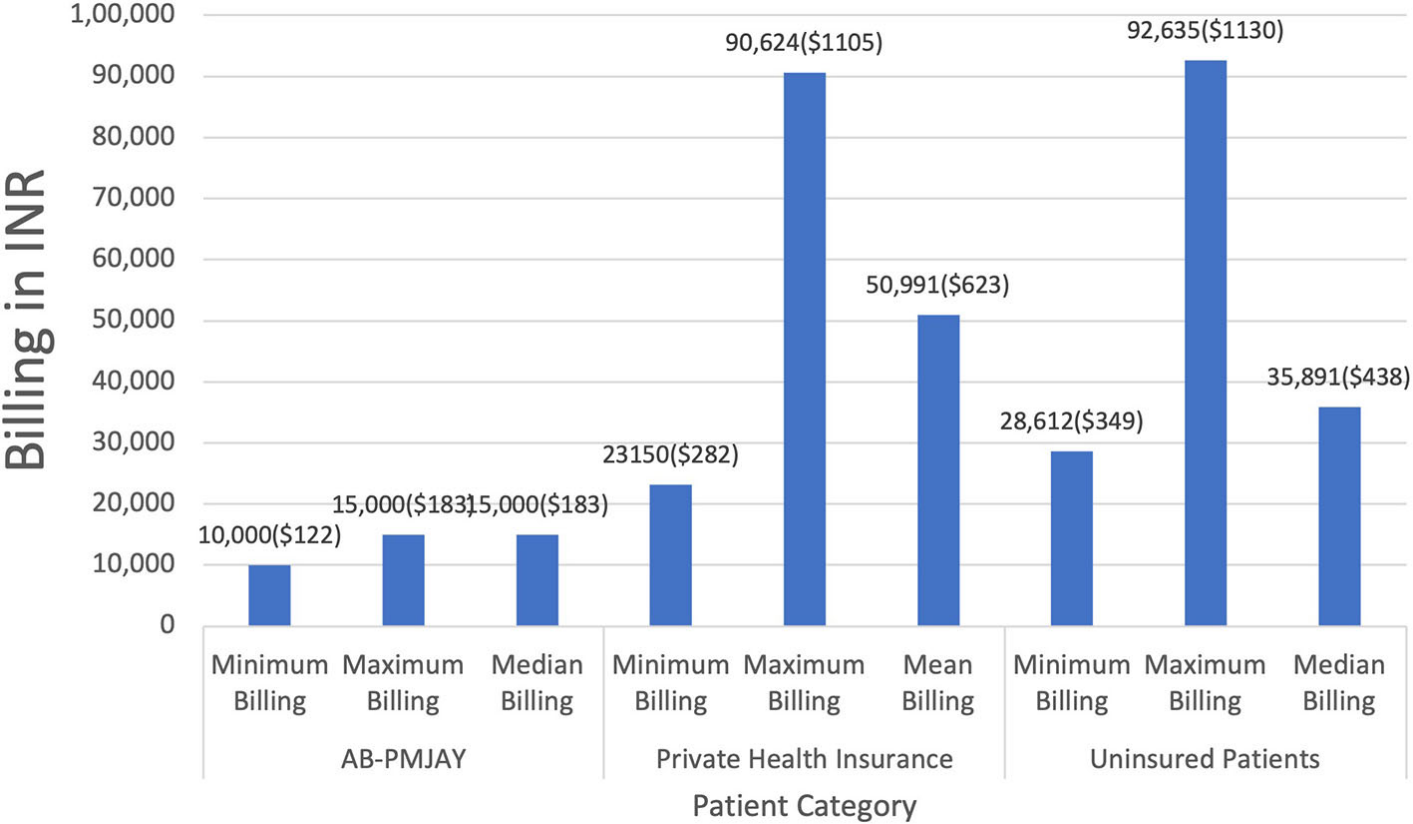
The figure shows the minimum, maximum, median, and mean billing amounts for Laparoscopic Cystectomy across AB-PMJAY, private insurance, and uninsured patients. Laparoscopic Cystectomy billing: Minimum, Maximum, Median and Mean. ^38^

Figure 5 presents the minimum, maximum, median and mean of billing amounts for Laparoscopic Cystectomy across AB-PMJAY, private health insurance and uninsured patients. AB-PMJAY had a minimum of Rs. 10,000 (122 USD) and a maximum billing of Rs. 15,000 (183 USD) with a median of Rs.15,000 (183 USD). Private health insurance ranged from a minimum of Rs.23,150 (282 USD) to a maximum of Rs.90,624 (1105 USD) with a mean of Rs.50,991 (623 USD). Uninsured patients ranged with a minimum billing of Rs. 28,612 (349 USD) to a maximum of Rs. 92,635 (1130 USD) and median of Rs.35,891(438 USD). Here median was used as the main statistic to measure the central tendency of AB-PMJAY and uninsured patients as the Shapiro-Wilk test yielded a p-value less than 0.05, indicating significant deviation from a normal distribution. Mean was used as the main statistic to measure the central tendency of private health insurance, as the Shapiro-Wilk test yielded a p-value of more than.005, indicating, normal distribution.

**Figure 6.**
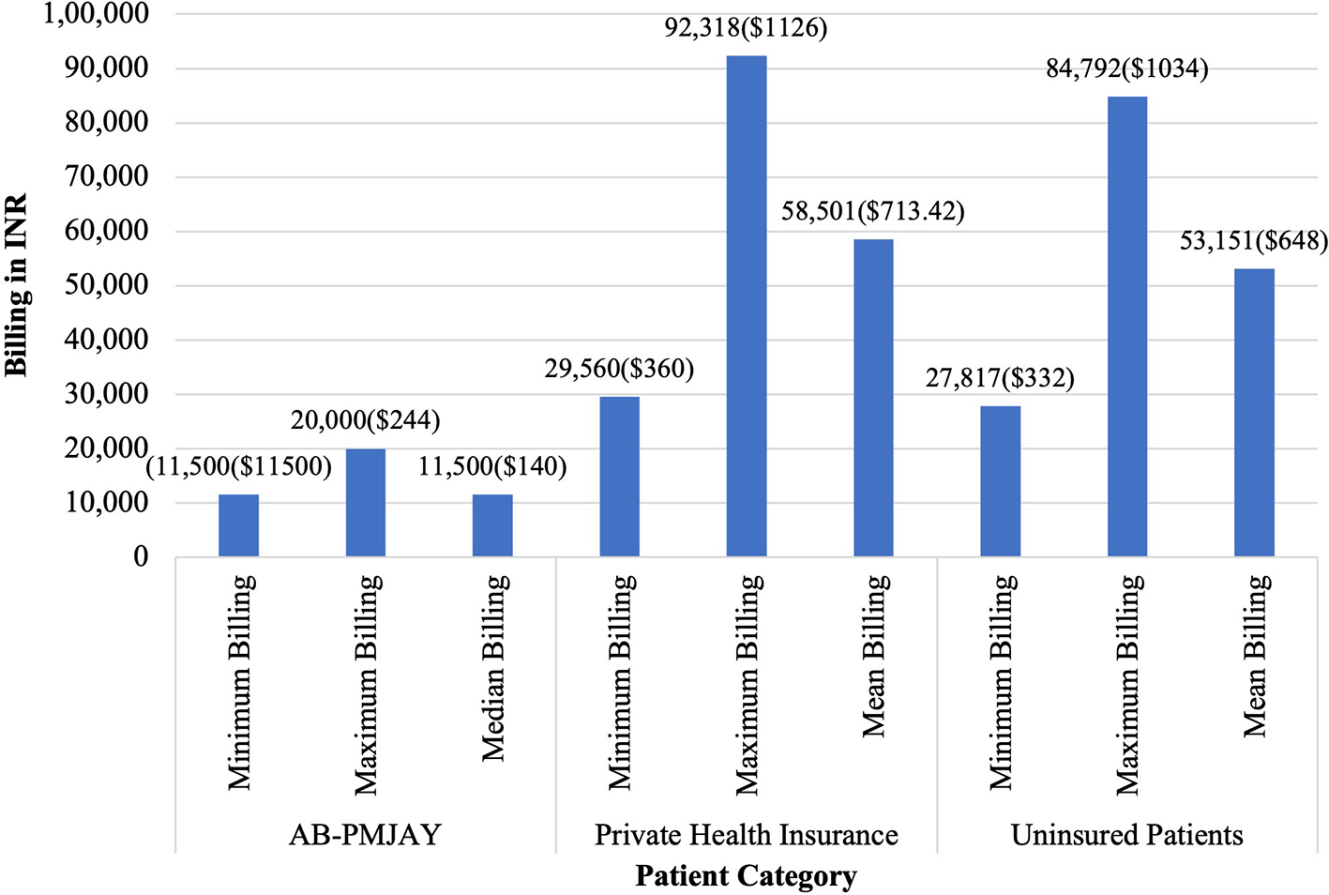
The figure shows the minimum, maximum, median, and mean billing amounts for LAVH across AB-PMJAY, private insurance, and uninsured patients. LAVH billing: Minimum, Maximum, Median and Mean. ^38^

Figure 6 presents the minimum, maximum, median and mean of billing amount for LAVH across AB-PMJAY, private health insurance and uninsured patients. AB-PMJAY had a minimum of Rs. 11,500 (140 USD) and a maximum billing of Rs. 20,000 (244 USD) with a median of Rs.11,500 (140 USD). Private health insurance ranged from a minimum of Rs.29,560 (360 USD) to a maximum of Rs.92,318 (1126 USD) with a mean of Rs.58,501 (713.42 USD). Uninsured patients ranged with a minimum billing of Rs. 27,187 (332 USD) to a maximum of Rs. 84,792 (1034 USD) and mean of Rs.53,151 (648 USD). Here mean was used as the main statistic to measure the central tendency of AB-PMJAY and uninsured patients as the Shapiro-Wilk test yielded a p-value of more than .05 for AB-PMJAY and uninsured patients, indicating a normal distribution. While median was used as the main statistic to measure the central tendency of private health insurance, as the Shapiro-Wilk test yielded a p-value less than.05, indicating significant deviation from a normal distribution.

### Variation in OOPE across various patient category

Table 4 presents variation in OOPE for C-section across AB-PMJAY, private health insurance and uninsured patients. All the 51 patients covered under AB-PMJAY had zero OOPE, as the scheme provided full coverage. Uninsured patients were most common with 531 cases, uninsured patients had 100% OOPE. The median OOPE for uninsured patients was Rs. 33,257. For patients under private health insurance, OOPE ranged widely depending on the insurance provider. Private health insurance had total 201 cases with a lower 38.15% average OOPE and median OOPE of ₹17,426 (212.51 USD) compared to uninsured patients. Star Health and Allied Insurance Co Ltd, Sampoorna Suraksha, Bajaj Allianz General Insurance Co. Ltd and Care Health Insurance Company Ltd had a average OOPE 68.35 %, 67.88 %, 58.47 % and 50.98 % respectively. While in Future Generali India Insurance Co Ltd, HDFC, GoDigit General Insurance Limited and Safeway TPA Pvt Ltd the average OOPE were 1.44 %, 7.15 %, 7.67 % and 8.53 % respectively. The mean OOPE in Bajaj Allianz General Insurance Co. Ltd, Manipal Foundation and Medicare were Rs. 1,16,559 (1421.45 USD), Rs. 54,651 (666.47 USD) and Rs. 50,695 (618.23 USD) respectively. The median OOPE in Medicare Patient, V Vidal Health TPA Pvt Ltd and Raksha Health Insurance TPA Pvt Ltd and Medi Assist India TPA Private Ltd were Rs. 32,377 (395 USD), Rs. 9678 (118 USD) and Rs. 9510 (116 USD) respectively.

**Table 4.** Variation in OOPE across patient category in C-Section. ^38^

Patient Category	Number of Cases	Total Percentage of Cases (%)	OOPE as average Percentage (%) of Billing Amount	Median OOPE/Mean OOPE *
**AB-PMJAY **	**51**	**5.64**	**0**	**0**
**Uninsured patients**	**531**	**58.67**	**100**	**33,257** ** **($405.57)**
**Private Health Insurances**	**201**	**22.21**	**38.15**	**17,426** ** **($212.51)**
Medi Assist India TPA Private Ltd	70	7.73	21.99	9,158 **($112)
Sampoorna Suraksha	43	4.75	67.88	27,744 **($338.34)
Medicare Patient	15	1.66	79	32,377 ** ($395)
ICICI Lombard General Insurance	13	1.44	32.91	7,033 ** ($86)
Medicare	11	1.22	41.3	50,695 *** ($618.23)
Raksha Health Insurance TPA Pvt Ltd	8	0.88	25.32	9,510 ** ($116)
Vidal Health TPA Pvt Ltd	8	0.88	39.57	9,578 ** ($117)
Paramount Health Services Insurance TPA Pvt Ltd	6	0.66	40.23	13,272 *** ($162)
Care Health Insurance Company Ltd	4	0.44	50.98	5,360 *** ($65.36)
Health Insurance TPA of India Ltd	3	0.33	38.35	15,935 *** ($194.32)
Star Health and Allied Insurance Co Ltd	3	0.33	68.35	31,492 *** ($384)
Bajaj Allianz General Insurance Co. Ltd	2	0.22	58.47	1,16,559 *** ($1421.45)
East West Assist Insurance TPA Pvt Ltd	2	0.22	50.95	6,384 *** ($78)
Health India	2	0.22	13.72	6,335 *** ($77.25)
IFFCO-Tokio General Insurance Company Ltd.	2	0.22	17.7	7,992 *** ($97.46)
Manipal Cigna - Suraksha Scheme	2	0.22	50.49	9,767 ***($119)
Manipal Foundation	2	0.22	45.91	54,651 ***($666.47)
Future Generali India Insurance Co Ltd	1	0.11	1.44	660 ***($8.04)
GoDigit General Insurance Limited	1	0.11	7.67	6,074 *** ($74)
HDFC	1	0.11	7.15	5,782 *** ($70.51)
Safeway TPA Pvt Ltd	1	0.11	8.53	6,997 ***($85.32)
United Healthcare India (P) LI	1	0.11	24.65	23,366 ***($285)

* Median and Mean OOPE in INR

** Median OOPE was considered to measure the central tendency when p-value was <0.05 in Shapiro-Wilk test, indicating significant deviation from a normal distribution. (e.g. Uninsured patients, ICICI Lombard).

*** Mean OOPE was considered when p-value was >0.05 in Shapiro-Wilk test, indicating normal distribution (e.g. Bajaj Allianz, Care Health Insurance).

**Figure 7.**
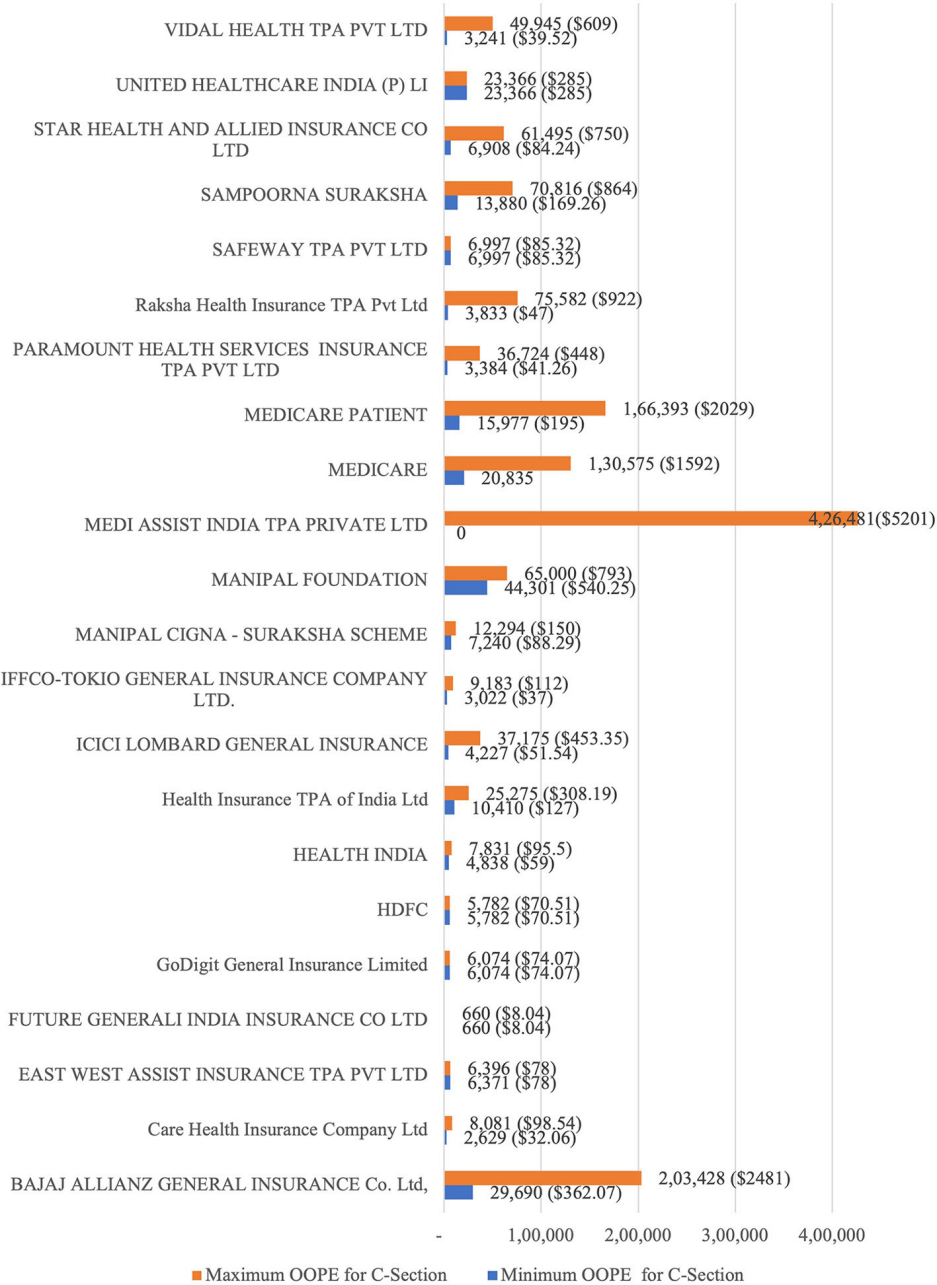
The figure shows the range of out-of-pocket expenses (OOPE) for C-sections across various private health insurances. C-Section Billing: Minimum and Maximum OOPE. ^38^ *Minimum and maximum OOPE in INR.

Figure 7 presents the variation in the minimum and maximum OOPE across different private health insurances for C-section. The OOPE range was widest for Bajaj Allianz (Rs. 29,690 [362 USD] - Rs. 2,03,428 [2481 USD]) and Medicare (Rs. 20,835 [254 USD] - Rs. 1,30,575 [1592 USD]). ICICI Lombard (Rs. 4,227[51.54 USD] - Rs. 37,175 [453.35 USD]), Paramount Health (Rs. 3,384 [41.26 USD] - Rs. 36,724 [448 USD]) and STAR Health (Rs. 6,908[84.24 USD] - Rs. 61,495[750 USD]) had more moderate range. Narrower OOPE ranges were seen for insurances Care Health (Rs. 2,629[32 USD] - Rs. 8,081[98.54 USD]) and East West Assist (Rs. 6,371[78 USD] - Rs. 6,396[78 USD]). Medi Assist India TPA Private Ltd was the only private health insurance that provided four patients out of 70 patients with zero OOPE.

**Figure 8.**
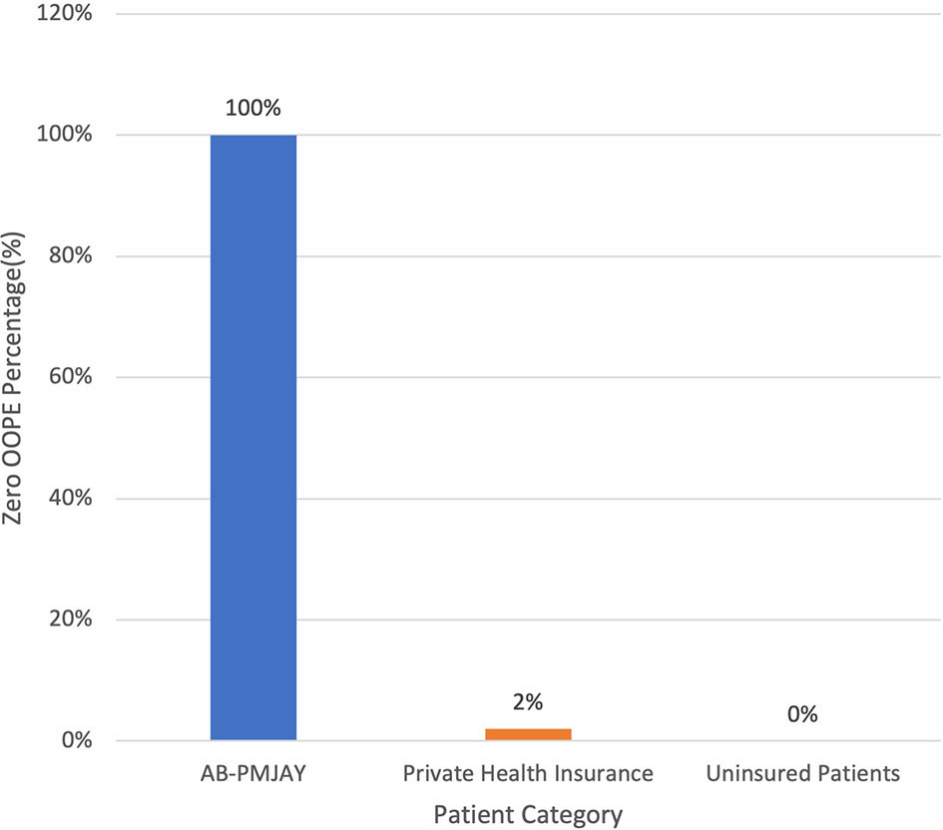
The figure shows the variation in the percentage of patients with zero out-of-pocket expenses (OOPE) for C-sections across different patient categories. Percentage of C-Section patients with zero OOPE across patient category. ^38^

Figure 8 presents the variation in percentage of patients with zero OOPE for C-section across patient category. 100% of AB-PMJAY beneficiaries had zero OOPE. However, only 2% of private insurance patients had zero OOPE. While none of the uninsured patients had zero OOPE.

Table 5 presents the variation in OOPE for laparoscopic hysterectomy across patient category: AB-PMJAY, private health insurance and uninsured patients. All the 11 patients under AB-PMJAY had zero OOPE. While all the 22 uninsured patients had 100% OOPE with median OOPE of Rs.57,053 (696 USD). For private health insurance patients, average percentage of OOPE varied across insurances, with patients paying 2.09-53.5 % on average. Sampoorna Suraksha and Star Health and Allied Insurance had the highest average percentage of OOPE of 53.5 % and 19.98% and also they had the highest mean OOPE of Rs. 36,605 (446.4 USD) and Rs. 23,709 (289.13 USD). Health Insurance TPA of India Ltd and Medi Assist India TPA Private Ltd had the lowest average percentage of OOPE of 2.09% and 4.91% and also they had lowest mean and median OOPE of Rs. 1,539 (19 USD) and Rs. 2,065 (25.18 USD).

**Table 5.** Variation in OOPE across Patient Category in Laparoscopic Hysterectomy. ^38^

Patient Category	Number of Cases	Total Percentage (%) of Cases	Average percentage (%) of OOPE	Median OOPE/Mean OOPE *
**AB-PMJAY **	**11**	**1.22**	**0**	**0**
**Uninsured patients**	**22**	**2.43**	**100**	**57,053** ** **($6960**
**Private Health Insurances**	**11**	**1.22**	**19.23**	**7,600** ** **($93)**
Medi Assist India TPA Private Ltd	04	0.44	4.91	2,065 **($25.18)
Sampoorna Suraksha	03	0.33	53.5	36,605 *** ($446.4)
Health Insurance TPA of India Ltd	02	0.22	2.09	1,539 ***($19)
Medicare Patient	01	0.11	7.64	7,615 *** ($93)
Star Health and Allied Insurance	01	0.11	19.98	23,709 *** ($289.13)

* Median and Mean OOPE in INR

** Median OOPE was considered to measure the central tendency when p-value in Shapiro-Wilk test was <0.05, indicating significant deviation from a normal distribution. (e.g. uninsured patients, Medi Assist India TPA Private Ltd).

*** Mean OOPE was considered p-value was >0.05 in Shapiro-Wilk test, indicating normal distribution (e.g. Sampoorna Suraksha, Health Insurance TPA of India Ltd).

**Figure 9.**
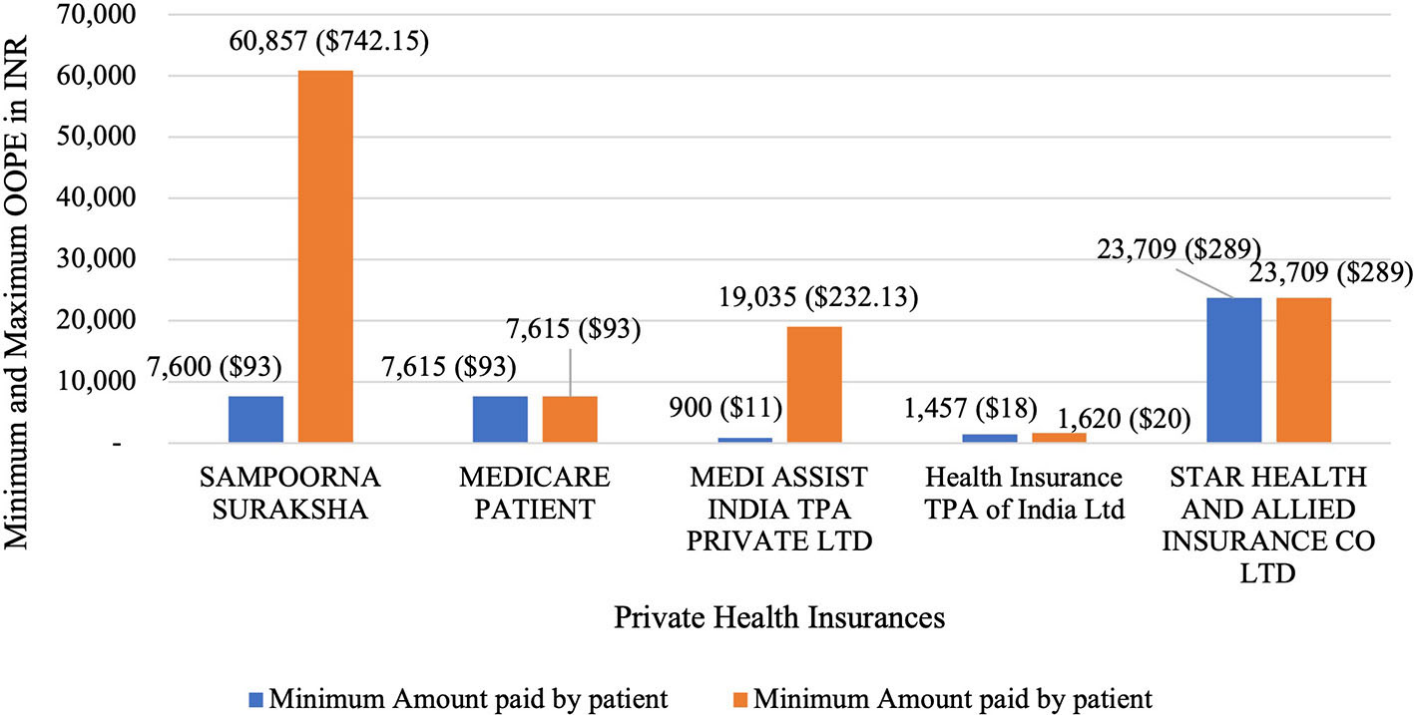
The figure shows OOPE for laparoscopic hysterectomy among different private health insurers. Minimum and Maximum OOPE for Different Private Health Insurances for Laparoscopic Hysterectomy. ^38^

Figure 9 present the variation in the minimum and maximum OOPE across private health insurances for laparoscopic hysterectomy. Sampoorna Suraksha had the widest range from Rs. 7,600 (93 USD) to Rs. 60,857 (742.15 USD). Medicare Patient and Star Health, with only one patient each, had OOPE of Rs. 7,615 (93 USD) and Rs. 23,709 (289.13 USD) respectively. Medi Assist India had a range from Rs. 900 (11 USD) to Rs. 19,035 (232.13 USD). While Health Insurance TPA of India had the narrowest range from Rs. 1,457 (18 USD) to Rs. 1,620 (20 USD).

**Figure 10.**
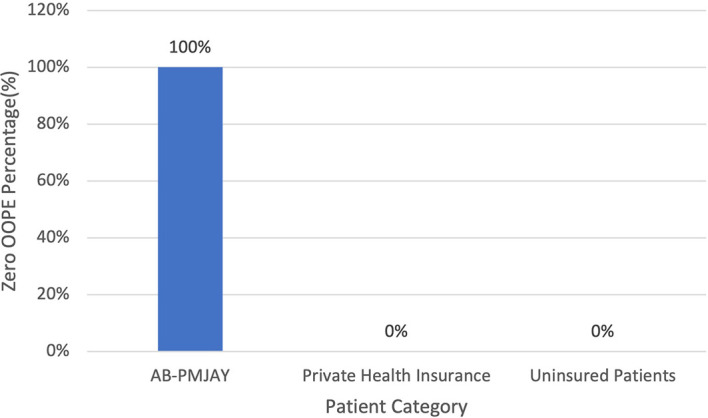
The figure shows the variation in the percentage of patients with zero out-of-pocket expenses (OOPE) for laparoscopic hysterectomy across different patient categories. Percentage of Laparoscopic Hysterectomy patients with zero OOPE across Patient Category. ^38^

Figure 10 present the variation in percentage of patients with zero OOPE for laparoscopic hysterectomy across patient category. 100% of AB-PMJAY beneficiaries had zero OOPE. While none of the patients under private health insurance and uninsured patients had zero OOPE for laparoscopic hysterectomy.

Table 6 presents the variation in OOPE for laparoscopic cystectomy across AB-PMJAY, private health insurance and uninsured patients. All the 7 patients under AB-PMJAY had zero OOPE. While all the 12 uninsured patients had 100% OOPE with median OOPE of Rs.35,891 (438 USD). For private insurance patients, average percentage of OOPE and median OOPE were 23.64% and Rs. 8,241 (100.5 USD). Medi Assist and MDIndia had the lowest average percentage of OOPE of 1.43% and 5.79%. Medicare Patient and Sampoorna Suraksha had high average percentage of OOPE of 36.79% and 29.7%. While Sampoorna Suraksha had the lowest median OOPE of Rs.4,880 (59.51 USD), Medicare Patient and Vidal Health TPA PVT LTD had highest mean OOPE of Rs.24,387 (297.4 USD) and Rs.12,850 (157 USD).

**Table 6.** Variation in OOPE across Patient Category in Laparoscopic Cystectomy. ^38^

Patient Category	Number of Cases	Total Percentage (%) of Cases	Average percentage (%) of OOPE	Median OOPE *
**AB-PMJAY **	**07**	**0.77**	**0**	**0**
**Uninsured patients**	**12**	**1.33**	**100**	**35,891** ** **($438)**
**Private Health Insurances**	**11**	**1.22**	**23.64**	**8,241** ** **($100.5)**
Sampoorna Suraksha	5	0.55	29.75	4,880 ** ($59.51)
Medicare Patient	02	0.22	36.79	24,387 *** ($297.4)
MDIndia Health Insurance TPA Pvt. Ltd	01	0.11	5.79	4,392 ***($53.56)
Medicare	01	0.11	12.26	11,119 *** ($135.59)
Medi Assist India TPA Private Ltd	01	0.11	1.43	850 ***($10.36)
Vidal Health TPA Pvt Ltd	01	0.11	18.16	12,850 *** ($157)

* Average Median and Mean OOPE in INR

** Median OOPE was considered to measure the central tendency when Shapiro-Wilk test p-value was <0.05, indicating significant deviation from a normal distribution. (e.g. uninsured patients and Sampoorna Suraksha).

*** Mean OOPE was considered when p-value was >0.05, indicating normal distribution (e.g. MDIndia Health Insurance TPA Pvt. Ltd and Medicare).

**Figure 11.**
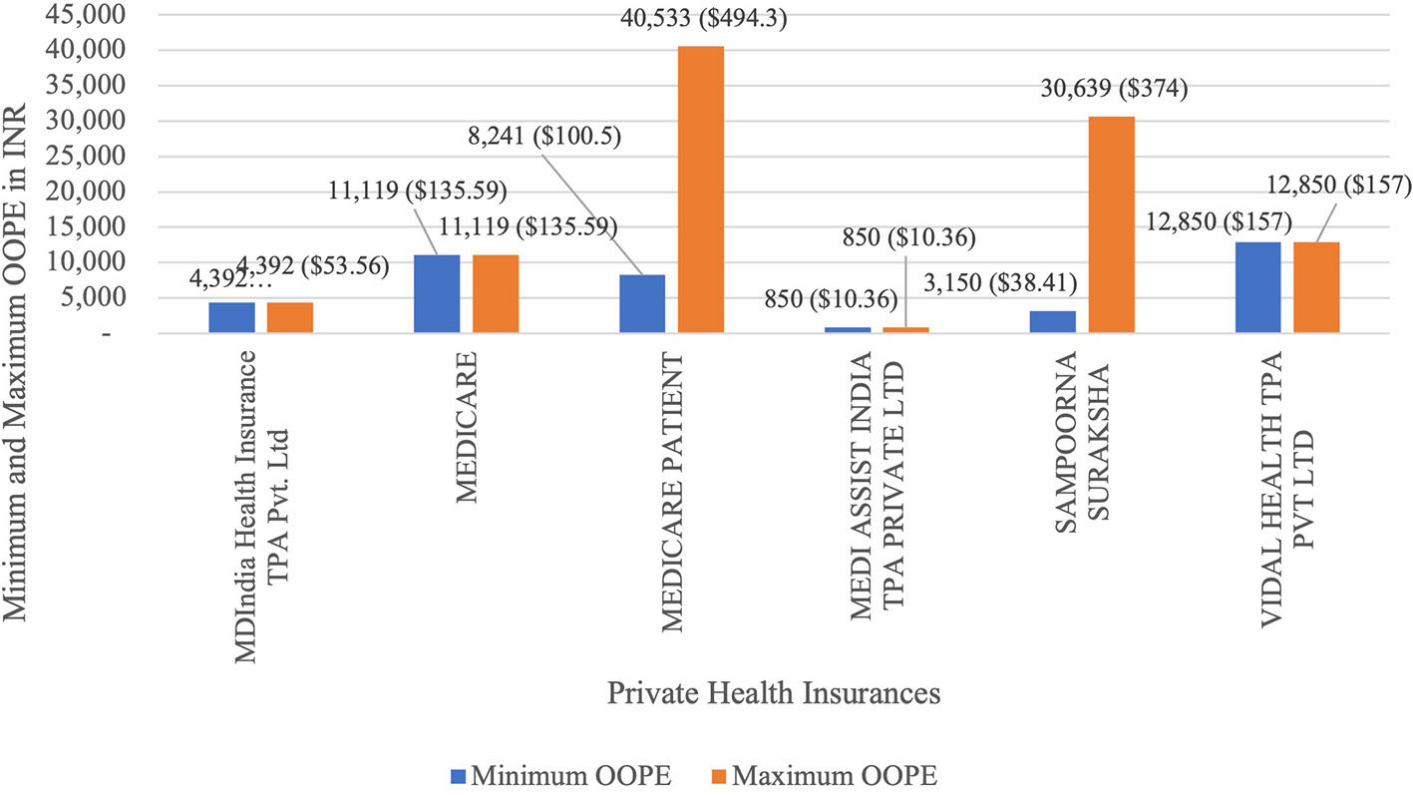
The figure shows the range of out-of-pocket expenses (OOPE) for laparoscopic cystectomy among different private health insurances. Minimum and Maximum OOPE for Different Private Health Insurances for Laparoscopic Cystectomy. ^38^

Figure 11 present the variation in the minimum and maximum OOPE across private health insurances for laparoscopic cystectomy. Medicare Patient had the widest range of OOPE, from Rs.8,241(100.5 USD) to Rs.40,533(494.3 USD). Sampoorna Suraksha had the from Rs.3,150(38.41 USD) to Rs.30,639(374 USD). MDIndia Health Insurance TPA Pvt. Ltd,Medicare, Medi Assist and Vidal Health had a maximum and minimum OOPE of Rs.4,392(53.56 USD), Rs.11,119(135.59 USD), Rs.850(10.36 USD) and Rs.12,850(157 USD) as each of them had only one case.

**Figure 12.**
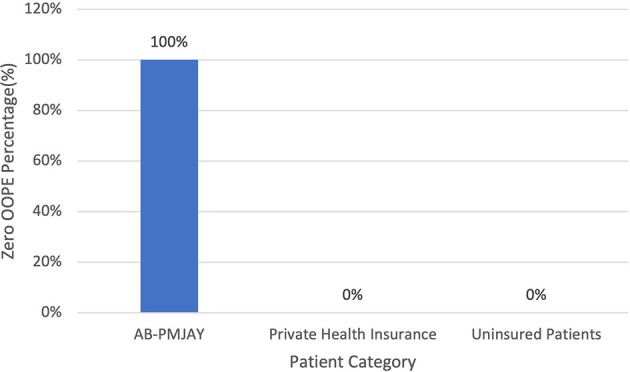
The figure shows the variation in the percentage of patients with zero out-of-pocket expenses (OOPE) for laparoscopic cystectomy across different patient categories. Percentage of Laparoscopic cystectomy patients with zero OOPE across patient category. ^38^

Figure 12 present the variation in percentage of patients with zero OOPE for laparoscopic cystectomy across patient category. 100% of AB-PMJAY beneficiaries had zero OOPE. While none of the patients under private health insurance and uninsured patients had zero OOPE for laparoscopic cystectomy.

Table 7 present the variation in OOPE for laparoscopic myomectomy across AB-PMJAY, private health insurance and uninsured patients. All the 6 patients under AB-PMJAY had zero OOPE. While all the 9 uninsured patients had 100% OOPE with mean OOPE of Rs.39,848 (486 USD). For private health insurance, the average percentage of OOPE was 18.58% with mean OOPE of Rs.5,435(66.28 USD). Sampoorna Suraksha and Medi Assist India TPA Private Ltd had average percentage of OOPE of 16.01% and 19.86% and mean OOPE of Rs.5,245(64 USD) and Rs.5,530(67.43 USD).

**Table 7.** Variation in OOPE across different patient category in Laparoscopic Myomectomy. ^38^

Patient Category	Number of Cases	Total Percentage (%) of Cases	Average percentage of OOPE	Median OOPE/Mean OOPE *
**AB-PMJAY **	**06**	**0.66**	**0**	**0**
**Uninsured patients**	**09**	**9.47**	**100**	**39,848** ** **($486)**
**Private Health Insurances**	**03**	**0.33**	**18.58**	**5,435** ** **($66.28)**
Sampoorna Suraksha	02	0.22	19.86	5,530 **($67.43)
Medi Assist India TPA Private Ltd	01	0.11	16.01	5,245 **($64)

* Median and Mean OOPE in INR

** Only Mean OOPE was considered to measure the central tendency as p-value was >0.05 in Shapiro-Wilk test, indicating normal distribution.

**Figure 13.**
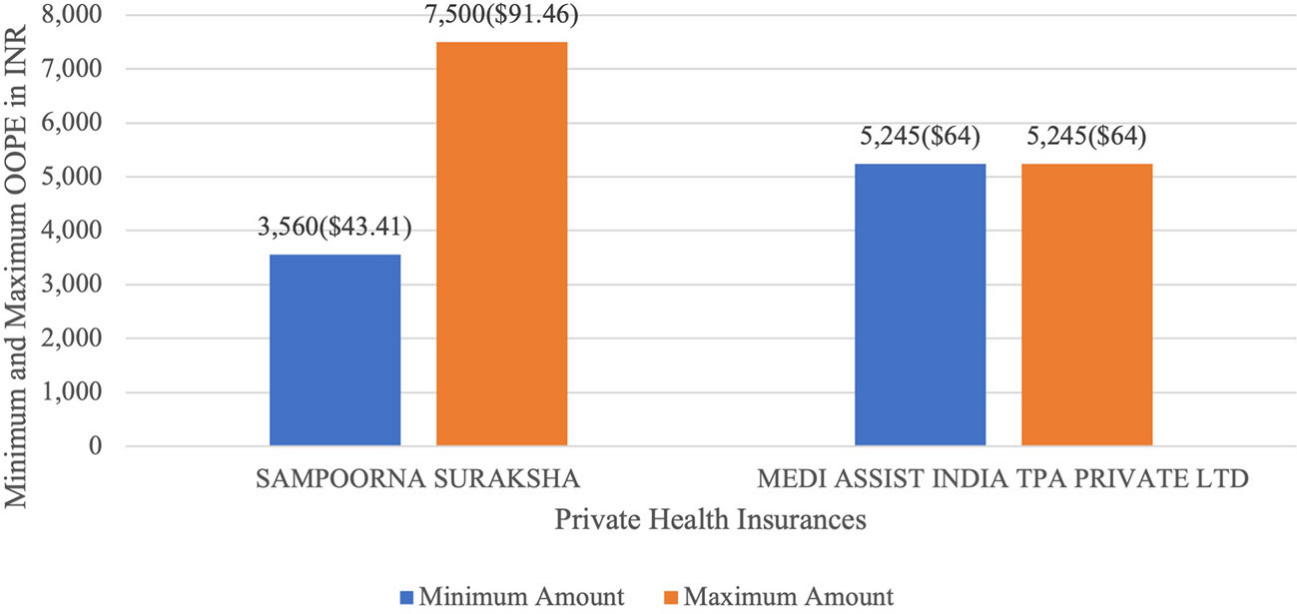
The figure shows the range of OOPE for laparoscopic myomectomy among different private health insurers. Variation in average OOPE across different patient category in Laparoscopic Myomectomy. ^38^

Figure 13 present the variation in the minimum and maximum OOPE across private health insurances for laparoscopic myomectomy. Private health insurances ranged from Rs.3,560 (43.41 USD)-Rs.7,500 (91.46 USD) for Sampoorna Suraksha and for Medi Assist India TPA Private Ltd the minimum and maximum OOPE was Rs.5,245(64 USD) as there was only one case of laparoscopic myomectomy.

**Figure 14.**
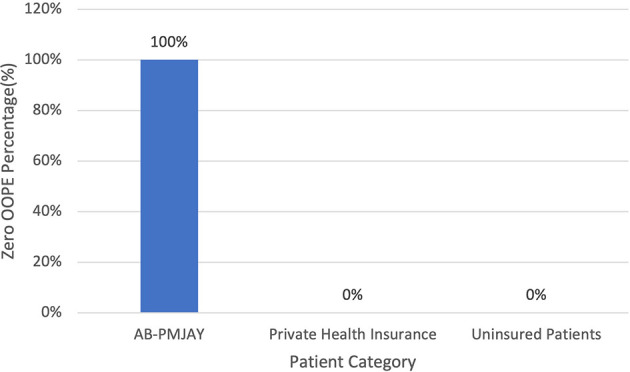
The figure shows the variation in the percentage of patients with zero out-of-pocket expenses (OOPE) for laparoscopic myomectomy across different patient categories. Percentage of Laparoscopic Myomectomy patients with zero OOPE across patient category. ^38^

Figure 14 present the variation in percentage of patients with zero OOPE for laparoscopic myomectomy across patient category. 100% of AB-PMJAY beneficiaries had zero OOPE. While none of the patients under private health insurance and uninsured patients had zero OOPE for laparoscopic myomectomy.

Table 8 present the variation in OOPE across AB-PMJAY, private health insurance and uninsured patients. All the 11 patients under AB-PMJAY had zero OOPE. While all the 13 uninsured patients had 100% OOPE with mean OOPE of Rs.53,151(648.18 USD). For private health insurance, the average percentage of OOPE was 15.05% with mean OOPE of Rs.7,230(88.17 USD). Under private health insurances, Sampoorna Suraksha had the highest average percentage of OOPE of 21.6% while Paramount Health Services Insurance TPA Pvt Ltd and Medi Assist India TPA Private Ltd had the lowest average OOPE of 4.39% and 10.28% and mean OOPE of Rs.3,758 (46 USD) and Rs.9,492 (116 USD).

**Table 8.** Variation in OOPE across different patient category for LAVH. ^38^

Patient Category	Number of Cases	Total Percentage (%) of Cases	Average percentage (%) of OOPE	Median OOPE/Mean OOPE *
**AB-PMJAY **	**11**	**1.22**	**0**	**0**
**Uninsured Patients**	**13**	**1.44**	**100**	**53,151** ** **($648.180**
**Private Health Insurance**	**06**	**0.66**	**15.05**	**7,230** ** **($88.17)**
Sampoorna Suraksha	03	0.33	21.6	7,368 ** ($90)
Paramount Health Services Insurance TPA Pvt Ltd	01	0.11	4.39	3,758 ** ($46)
Medi Assist India TPA Private Ltd	01	0.11	10.28	9,492 ** ($116)
Star Health and Allied Insurance Co Ltd	01	0.11	10.82	8,022 **($98)

* Median and Mean OOPE in INR

** Only Mean OOPE was considered to measure the central tendency as p-value was >0.05 in Shapiro-Wilk test, indicating normal distribution.

**Figure 15.**
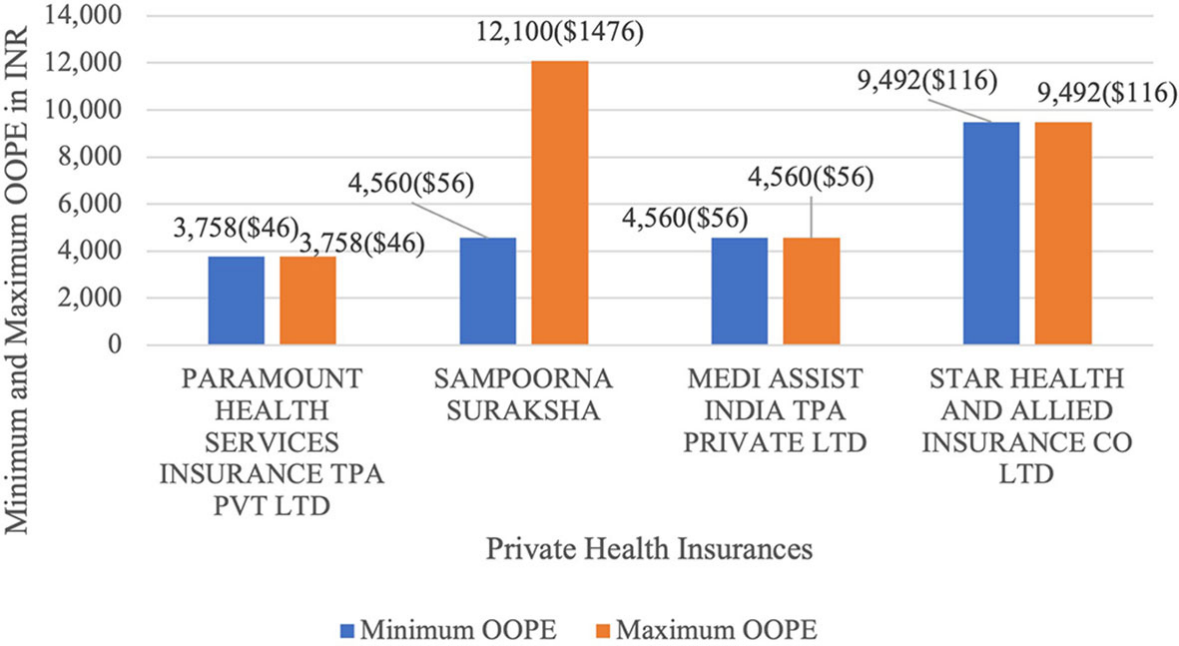
The figure shows the range of out-of-pocket expenses (OOPE) for LAVH among different private health insurers. Minimum and Maximum OOPE for Different Private Health Insurances for LAVH. ^38^

Figure 15 present the variation in the minimum and maximum OOPE across private health insurances for LAVH. Sampoorna Suraksha ranged from Rs.4,560 (56 USD) minimum up to Rs.1,21,000 (1476 USD) maximum. While Paramount Health Services Insurance TPA Pvt Ltd, Medi Assist India TPA Private Ltd and Star Health and Allied Insurance Co Ltd had the minimum and maximum OOPE of Rs.3,758 (46 USD), Rs.4560 (56 USD) and Rs.9,492 (116 USD) as there were only one case of LAVH for each of the insurances.

**Figure 16.**
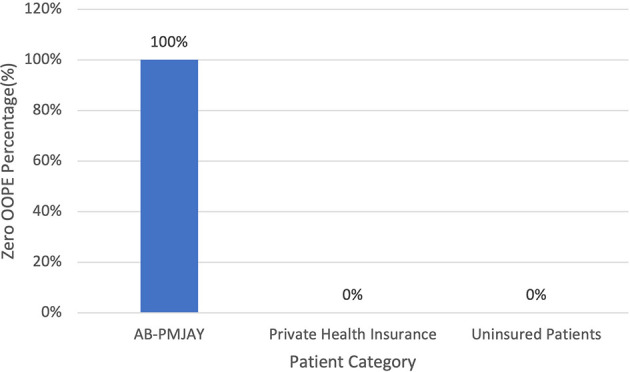
The figure shows the variation in the percentage of patients with zero out-of-pocket expenses (OOPE) for LAVH across different patient categories. Percentage of LAVH patients with zero OOPE by patient category. ^38^

Figure 16 present the variation in percentage of patients with zero OOPE for LAVH across patient category. 100% of AB-PMJAY beneficiaries had zero OOPE. While none of the private health insurance patients and uninsured patients had zero OOPE for LAVH.

### Average and median OOPE of the selected surgeries

**Figure 17.**
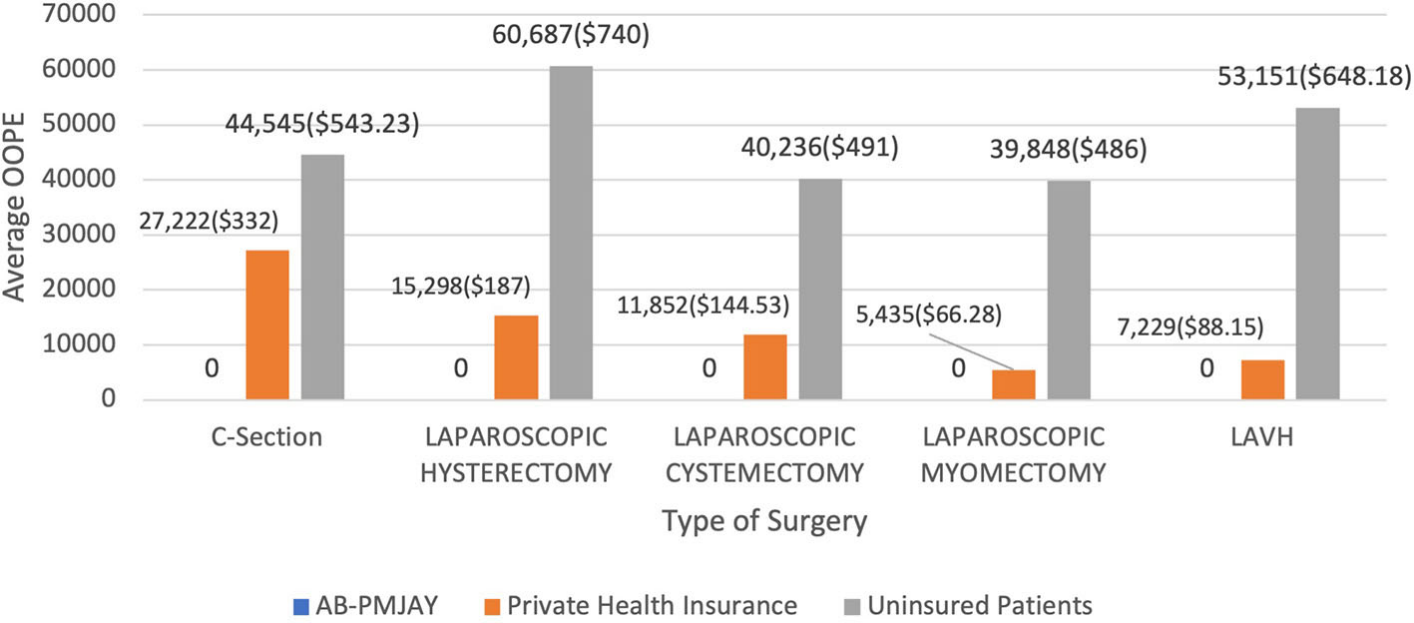
The figure presents the average out-of-pocket expenses (OOPE) for C-Section, Laparoscopic Hysterectomy, Laparoscopic Cystectomy, Laparoscopic Myomectomy, and LAVH across AB-PMJAY, private health insurance, and uninsured patients. Average OOPE for selected surgeries across patient category. ^38^

Figure 17 presents the average OOPE in C-Section, Laparoscopic Hysterectomy, Laparoscopic Cystectomy, Laparoscopic Myomectomy and LAVH across AB-PMJAY, Private Health Insurance and uninsured patients. Patients covered under AB-PMJAY incurred zero OOPE for C-Section, Laparoscopic Hysterectomy, Laparoscopic Cystectomy, Laparoscopic Myomectomy and LAVH. Patients under private health insurance had an average OOPE of Rs.27,222 (332 USD), Rs.15,298 (187 USD), Rs.11,852 (144.53 USD), Rs.5,435 (66.28 USD) and Rs.7,229 (88.15 USD) for C-Section, Laparoscopic Hysterectomy, Laparoscopic Cystectomy, Laparoscopic Myomectomy and LAVH. For C-Section, Laparoscopic Hysterectomy, Laparoscopic Cystectomy, Laparoscopic Myomectomy and LAVH patients uninsured patients had higher financial burden, with average OOPE of Rs.44,545 (543.23 USD), Rs.60,687 (740 USD), Rs.40,236 (491 USD), Rs.39,848 (486 USD) and Rs.53,151 (648.18 USD).

**Figure 18.**
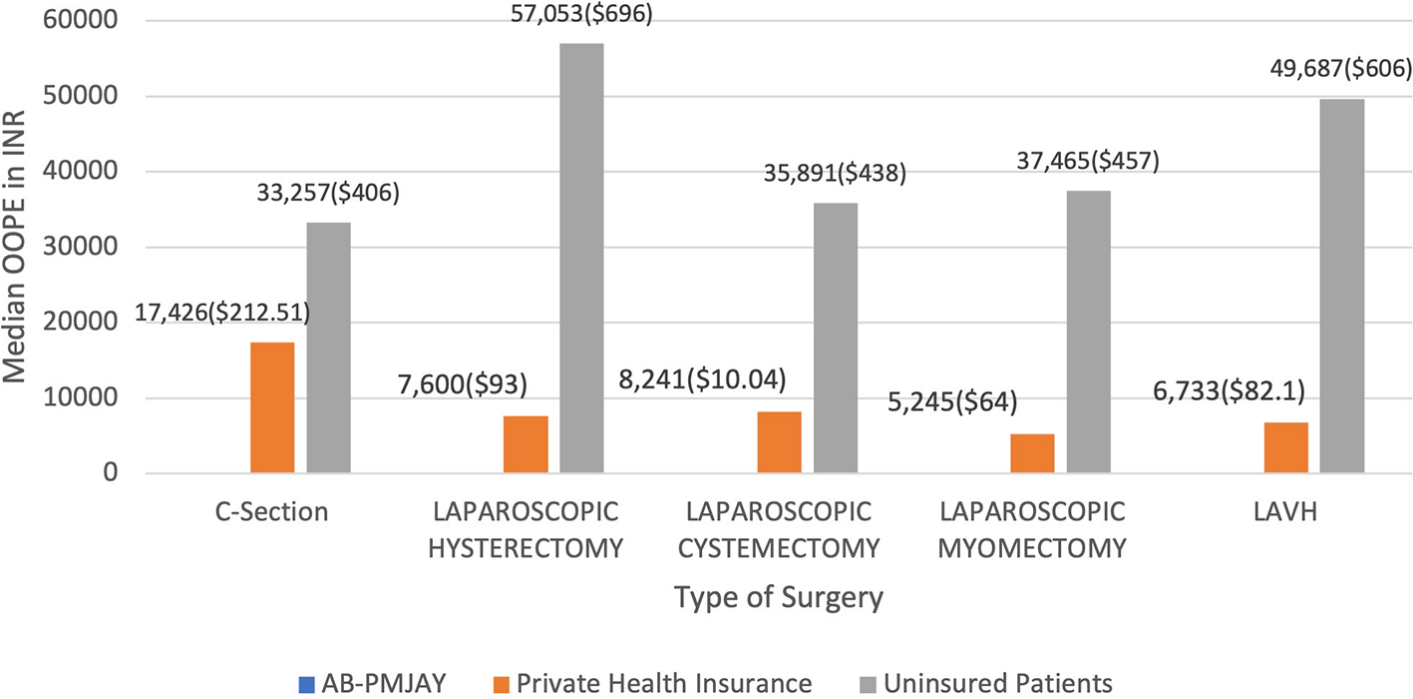
The figure presents the median out-of-pocket expenses (OOPE) for C-Section, Laparoscopic Hysterectomy, Laparoscopic Cystectomy, Laparoscopic Myomectomy, and LAVH across AB-PMJAY, private health insurance, and uninsured patients. Median OOPE for selected surgeries across patient category. ^38^

Figure 18 present the median OOPE in C-Section, Laparoscopic Hysterectomy, Laparoscopic Cystectomy, Laparoscopic Myomectomy and LAVH across AB-PMJAY, Private Health Insurance and uninsured patients. Patients covered under AB-PMJAY had zero OOPE for C-Section, Laparoscopic Hysterectomy, Laparoscopic Cystectomy, Laparoscopic Myomectomy and LAVH. Patients under private health insurance had median OOPE of Rs.17,426 (212.51 USD), Rs.7,600 (93 USD), Rs.8,241(10.04 USD), Rs.5,245(64 USD) and Rs.6,733(82.1 USD) for C-Section, Laparoscopic Hysterectomy, Laparoscopic Cystectomy, Laparoscopic Myomectomy and LAVH. For C-Section, Laparoscopic Hysterectomy, Laparoscopic Cystectomy, Laparoscopic Myomectomy and LAVH patients who were uninsured had higher financial burden, with median OOPE of Rs.33,257 (406 USD), Rs.57,053 (696 USD), Rs.35,891 (438 USD), Rs.37,456 (457 USD) and Rs.49,687 (606 USD)

## Discussion

There is little existing peer reviewed literature on OOPE among obstetrics and gynaecology patients. The current study offers valuable insights into the OOPE incurred by obstetrics and gynaecology patients undergoing various surgical procedures (C-section, laparoscopic hysterectomy, laparoscopic cystectomy, laparoscopic myomectomy and LAVH) across AB-PMJAY, private health insurance and uninsured patients. C-Section, laparoscopic hysterectomy, laparoscopic cystectomy, laparoscopic myomectomy and LAVH were the top five most performed obstetrics and gynecology surgeries in the tertiary care teaching hospital between January 2023 and July 2023 with a total of 783 C-Section cases, 44 cases of Laparoscopic Hysterectomy, 30 cases of Laparoscopic Cystectomy, 18 cases of Laparoscopic Myomectomy and 30 cases of LAVH.

The study demonstrates the effectiveness of AB-PMJAY in reducing OOPE and enhancing financial risk protection for obstetrics and gynaecology patients. 100% of AB-PMJAY beneficiaries incurred zero OOPE across all analyzed surgical procedures. While private health insurance lowered OOPE compared to uninsured patients, patients still faced substantial OOPE burdens. This indicates that private insurance may not adequately cover the complete costs of these surgical procedures for obstetrics and gynaecology patients like government-sponsored health insurance schemes. ^27, 28^

The variation in OOPE across the groups can be attributed to several factors. For AB-PMJAY beneficiaries, the zero OOPE can be explained by the comprehensive coverage provided by the government scheme, which fully reimburses healthcare costs, thus removing any financial burden from patients. ^28^ On the other hand, uninsured patients face the full brunt of medical expenses, leading to substantial OOPE, which often results in financial strain and potentially catastrophic health costs. ^26^

Private health insurance, while alleviating some costs, does not provide complete coverage for all the expenses incurred during surgeries. This is often due to factors like co-payments, restrictions on bed categories, or incomplete coverage of certain medical services. As a result, private insurance patients still experience significant OOPE, althoug lower than that faced by uninsured patients. ^29^ These differences highlight that while private health insurance can reduce OOPE, it does not offer the same level of protection as government schemes like AB-PMJAY. ^28^

For obstetrics and gynaecology patients OOPE remains a critical barrier to timely and quality healthcare, particularly for disadvantaged socioeconomic groups. ^30, 31^ By alleviating OOPE burdens, AB-PMJAY facilitates access to affordable and accessible obstetrics and gynaecology surgeries for such populations. This has the potential to mitigate maternal mortality rates associated with financial constraints preventing access to obstetric care. ^32, 33^

As shown in Table 2, the analysis revealed that the average and median OOPE under AB-PMJAY was zero across all analyzed surgeries. Conversely, uninsured patients had substantial median OOPE, ranging from Rs. 33,257 (406 USD) for C-section to Rs. 57,053 (696 USD) for laparoscopic cystectomy. Such high OOPE can potentially lead to financial strain and adverse health outcomes. ^26, 34^ The variation in OOPE across different private health insurance plans suggests differences in bed categories, individualized benefit plans and co-payments. Median OOPE under private insurance was also significant, varying from Rs. 5,245 (64 USD) for laparoscopic myomectomy to Rs. 17,426 (212.5 USD) for C-section. These findings align with previous observations concerning private insurance coverage limitations. ^29^

High OOPE presents a significant barrier to achieving universal health coverage and equitable access to quality healthcare services. ^35^ Expanding government-funded insurance initiatives like AB-PMJAY is critical to mitigating inequities in obstetrics and gynaecology healthcare utilization and OOPE reduction. ^23, 36, 37^ Such efforts can alleviate financial barriers and promote greater participation in the healthcare system.

The study’s sample size restricted to a single tertiary care teaching hospital limits the generalizability of findings. Future research with larger, diverse samples could provide deeper insights into variations in OOPE based on geographical location, healthcare infrastructure and population demographics.

## Ethical approval and consent

The study was conducted following the approval of the ethics committee of the study setting. As the study is retrospective in nature, patient consent was not required. No identifiable images or data of individuals were used in the study.

## Ethical consideration

Institutional Ethics Committee (IEC) approval was granted from the Kasturba Medical College and Kasturba Hospital Institutional Ethics Committee – 2: DHR registration no. EC/NEW/INST/2021/1707 (IEC2:562/2023) on 19-1-24. The patient data was made anonymous and treated with strict confidentiality for the study. As the study is a retrospective study and did not involve any new treatments or interventions, informed consent was not applicable. Consent was waived by the ethics committee.

## Data Availability

Figshare: Out-of-Pocket Expenditure (OOPE) on Selected Surgeries in the Obstetrics and Gynaecology Department incurred by Ayushman Bharat Pradhan Mantri Jan Arogya Yojana (AB-PMJAY), Private Health Insurance and Uninsured Patients in a Tertiary Care Teaching Hospital in Karnataka state of India. https://doi.org/10.6084/m9.figshare.27080365.v3. ^38^ The project contains the following underlying data:
• OOPE_OBG.xlsx (All the billing amounts for patients under the AB-PMJAY scheme, uninsured patients, and those covered by private health insurance are anonymized. No personal identifying information is included in the dataset). and Proforma.pdf (All patient billing details for AB-PMJAY, private health insurance and uninsured patients were collected via this validated proforma). Data are available under the terms of the Creative Commons Attribution 4.0 International license (CC-BY 4.0). Fihsare: Out-of-Pocket Expenditure (OOPE) on Selected Surgeries in the Obstetrics and Gynaecology Department incurred by Ayushman Bharat Pradhan Mantri Jan Arogya Yojana (AB-PMJAY), Private Health Insurance and Uninsured Patients in a Tertiary Care Teaching Hospital in Karnataka state of India. https://doi.org/10.6084/m9.figshare.27080365.v3. ^38^ The project contains the following data:
• Proforma.pdf Data is licensed under CC BY 4.0.

## References

[1] Press Information Bureau: The share of Out-of-Pocket expenditure in total health expenditure has reduced from 62.6% to 47.1%. 25 APR 2023. Reference Source

[2] Ministry of Health and Family Welfare: NHA Estimates 2019-2020. 25 APR 2023. Reference Source

[3] Insurance Regulatory and Development Authority of India (IRDAI): Annual Report 2021-22. 8 APR 2022. Reference Source

[4] Insurance Regulatory and Development Authority of India: *Annual report 2017-2018* [09 JAN 2019]. Reference Source

[5] National Health Authority: PMJAY states at a glance. Reference Source

[6] Employees’ State Insurance Corporation: Annual report 2018-2019. [31 MAR. 2019]. Reference Source

[7] Ministry of Health and Family Welfare: CGHS dashboard. 2021. Reference Source

[8] Sarwal R Kumar A : Health Insurance for India’s Missing Middle. 2021 Oct 27. 10.31219/osf.io/s2x8r

[9] Press Information Bureau: Ayushman Bharat: Providing Holistic Healthcare in Karnataka (State Series). 20 MAR 2023. Reference Source

[10] Press Information Bureau: Significant Decline in Maternal Mortality in India. 14 Dec 2022. Reference Source

[11] Sample Registration System (SRS): Special Bulletin On Maternal Mortality In India 2018-20. 28 Nov 2022. Reference Source

[12] Government of Meghalaya: The Meghalaya Health Policy 2021. 2021. Reference Source

[13] Hamal M Dieleman M De Brouwere V : Social determinants of maternal health: a scoping review of factors influencing maternal mortality and maternal health service use in India. *Public Health Rev.* 2020;41(1):13. 10.1186/s40985-020-00125-6 32514389 PMC7265229

[14] Paul P Chouhan P : Socio-demographic factors influencing utilization of maternal health care services in India. *Clinical Epidemiology and Global Health.* 2020 Sep;8(3):666–670. 10.1016/j.cegh.2019.12.023

[15] Ministry of Health and Family Welfare (MoHFW): Health and Family Welfare Statistics in India 2019-20. Reference Source

[16] Shekhar C Paswan B Singh A : Prevalence, sociodemographic determinants and self-reported reasons for hysterectomy in India. *Reprod. Health.* 2019;16:118. 10.1186/s12978-019-0780-z 31375139 PMC6679457

[17] Desai S Sinha T Mahal A : Prevalence of hysterectomy among rural and urban women with and without health insurance in Gujarat, India. *Reprod. Health Matters.* 2011;19(37):42–51. 10.1016/S0968-8080(11)37553-2 21555085

[18] Onoh RC Ezeonu PO Lawani LO : Experiences and challenges of gynecological endoscopy in a low-resource setting, Southeast Nigeria. *Trop. J. Obstet. Gynaecol.* 2018;35:30–37. 10.4103/TJOG.TJOG_34_17

[19] ComfortAB PetersonLA HattLE : Effect of health insurance on the use and provision of maternal health services and maternal and neonatal health outcomes: a systematic review. *J. Health Popul. Nutr.* 2013;31(4 Suppl 2):S81–S105.24992805

[20] Sanogo NA Yaya S : Wealth Status, Health Insurance, and Maternal Health Care Utilization in Africa: Evidence from Gabon. *Biomed. Res. Int.* 2020;2020:4036812–4036830. 10.1155/2020/4036830 32461984 PMC7212326

[21] Rashad AS Sharaf MF Mansour EI : Does public health insurance increase maternal health care utilization in Egypt? Frankfurt School - Working Paper Series. 2016. No. 223. Reference Source

[22] Yaya S Da F Wang R : Maternal healthcare insurance ownership and service utilisation in Ghana: Analysis of Ghana Demographic and Health Survey. *PLoS ONE.* 2019;14(4):e0214841. 10.1371/journal.pone.0214841 31022201 PMC6483336

[23] Sahoo J Singh SV Gupta VK : Do socio-demographic factors still predict the choice of place of delivery: A cross-sectional study in rural North India. *J Epidemiol Glob Health.* 2015;5(4 Suppl 1):S27–S34. 10.1016/j.jegh.2015.05.002 26073573 PMC7325830

[24] Olonade O Olawande TI Alabi OJ : Maternal Mortality and Maternal Health Care in Nigeria: Implications for Socio-Economic Development. *Open Access Maced. J. Med. Sci.* 2019 Mar 15;7(5):849–855. 10.3889/oamjms.2019.041 30962850 PMC6447322

[25] Miteniece E Pavlova M Shengelia L : Barriers to accessing adequate maternal care in Georgia: a qualitative study. *BMC Health Serv. Res.* 2018;18:631. 10.1186/s12913-018-3432-z 30103763 PMC6090778

[26] Ghosh S : Catastrophic payments and impoverishment due to out-of-pocket health spending in India. *Econ. Polit. Wkly.* 2014.

[27] Sood N : Government health insurance for people below poverty line in India: quasi-experimental evaluation of insurance and health outcomes. *BMJ (Clinical research ed.).* 11 Sep. 2014;349:g5114. 10.1136/bmj.g5114 25214509 PMC4161676

[28] Nandi S Schneider H Dixit P : Hospital utilization and out-of-pocket expenditure in public and private sectors under the universal government health insurance scheme in Chhattisgarh State, India: Lessons for universal health coverage. *PLOS ONE.* 2017 Nov;12(11):e0187904. 10.1371/journal.pone.0187904 29149181 PMC5693461

[29] Wu R Runguo : The effects of private health insurance on universal health coverage objectives in China: A systematic literature review. *Int. J. Environ. Res. Public Health.* 2020 Mar 19;17(6):2049. 10.3390/ijerph17062049 32204527 PMC7142974

[30] Mohanty SK Srivastava A : Out-of-pocket expenditure on institutional delivery in India. *Health Policy Plan.* 2013 May;28(3):247–262. 10.1093/heapol/czs057 22709923

[31] Bonu S : Incidence and correlates of catastrophic out-of-pocket health expenditures in India: evidence from the national sample survey. *BMJ.* 2009.

[32] Owusu PA Sarkodie SA Pedersen PA : Relationship between mortality and health care expenditure: Sustainable assessment of health care system. *PLoS ONE.* 2021 Feb;16(2):e0247413. 10.1371/journal.pone.0247413 33626059 PMC7904168

[33] Kumar S : Reducing maternal mortality in India: Policy, equity and quality issues. *Indian J. Public Health.* 2010;54:57–64. 10.4103/0019-557X.73271 21119236

[34] Bhojani U Thriveni BS Devadasan R : Out-of-pocket healthcare payments on chronic conditions impoverish urban poor in Bangalore, India. *BMC Public Health.* 2012 Dec;12(1):1–4. 10.1186/1471-2458-12-990 23158475 PMC3533578

[35] Manthan A : Roadmap for Universal Health Coverage in India. *ABDM.Gov.in.* 2022 [cited 2024 Jan 24].

[36] Limwattananon S Tangcharoensathien V Tisayaticom K : Why has the Universal Coverage Scheme in Thailand achieved a pro-poor public subsidy for health care?. *BMC Public Health.* 2012;12. 10.1186/1471-2458-12-S1-S6 22992431 PMC3382631

[37] Powell-Jackson T Hanson K : Financial incentives for maternal health: Impact of a national program in Nepal. *J. Health Econ.* 2012;31:271–284. 10.1016/j.jhealeco.2011.10.010 22112695

[38] Kamath S Dr. Sankar Acharya S Dr. Brand H : Out-of-Pocket Expenditure (OOPE) on Selected Surgeries in the Obstetrics and Gynaecology Department incurred by Ayushman Bharat Pradhan Mantri Jan Arogya Yojana (AB-PMJAY), Private Health Insurance and Uninsured Patients in a Tertiary Care Teaching Hospital in Karnataka state of India. figshare. *Journal contribution.* 2024. 10.6084/m9.figshare.27080365.v3 PMC11250400 39015849

