# Supplementary material for: Out-of-Pocket Expenditure (OOPE) on Selected Surgeries in the Obstetrics and Gynaecology Department incurred by Ayushman Bharat Pradhan Mantri Jan Arogya  Yojana (AB-PMJAY), Private Health Insurance and Uninsured Patients in a Tertiary Care Teaching Hospital in Karnataka state of India
Source: F1000Res. 2025 Jan 28;13:1511. Originally published 2024 Dec 9. [Version 2] doi: 10.12688/f1000research.157203.2 (PMC11807242; doi:10.12688/f1000research.157203.2)
Supplement: Peer review history

# Reviewer report: Reviewer response for version 2

**DOI:** 10.5256/f1000research.176681.r363071

**Published:** 2025-02-08

**Authors:** Risky Kusuma Hartono

**Affiliations:** 1 Department of Public Health, Universitas Indonesia Maju, Jakarta, Indonesia

Thank you for sending the revised version of the article. I have reviewed the changes made and the revisions are quite good. There have been significant improvements, and the article is now in a much better position for indexing.

Is the work clearly and accurately presented and does it cite the current literature?

Partly

If applicable, is the statistical analysis and its interpretation appropriate?

Partly

Are all the source data underlying the results available to ensure full reproducibility?

Yes

Is the study design appropriate and is the work technically sound?

Yes

Are the conclusions drawn adequately supported by the results?

No

Are sufficient details of methods and analysis provided to allow replication by others?

No

Reviewer Expertise:

Health Economic, health insurance, and disesase prevention

I confirm that I have read this submission and believe that I have an appropriate level of expertise to confirm that it is of an acceptable scientific standard.

# Reviewer report: Reviewer response for version 1

**DOI:** 10.5256/f1000research.172621.r351231

**Published:** 2025-01-02

**Authors:** Risky Kusuma Hartono

**Affiliations:** 1 Department of Public Health, Universitas Indonesia Maju, Jakarta, Indonesia

**Introduction:**

1. There is insufficient emphasis on the importance of selecting specific types of operations to be analyzed or researched.
2. The study does not address the local context in Karnataka, particularly the geographical or socio-economic differences that may influence OOPE (Out-of-Pocket Expenditure).
3. Not yet linking the impact of OOPE with catastrophic conditions that can have an impact on poverty

**Method:**

1. There is no clear definition of OOPE in the method and other variables that significant influencing the OOPE incident
2. Analysis using econometric and multivariate approaches will provide a more detailed description of the characteristics of patients who are more susceptible to OOPE.

**Results:**

1. The graphs should have more in-depth interpretation.
2. Too many graph, maybe some of them can be combined or adjusted
3. There is insufficient emphasis on the social impact, particularly on uninsured patients.

**Discussion:**

1. The discussion is overly focused on the data presented without comparing it to previous studies.
2. The discussion merely reiterates the results without analyzing the reasons behind differences in OOPE among groups.
3. It does not address the impact of OOPE on patients.
4. There is no detailed explanation of whether other factors, such as geographical location, educational level, or type of healthcare facility, influenced the results.
5. There is no clear explanation of how these findings can influence policymakers, insurance providers, or hospitals.

**Conclusion:**

1. The study does not provide a conclusion summarizing the research findings.
2. There are no specific recommendations for health insurance companies or hospitals to prevent OOPE in patients in the future.

Is the work clearly and accurately presented and does it cite the current literature?

Partly

If applicable, is the statistical analysis and its interpretation appropriate?

Partly

Are all the source data underlying the results available to ensure full reproducibility?

Yes

Is the study design appropriate and is the work technically sound?

Yes

Are the conclusions drawn adequately supported by the results?

No

Are sufficient details of methods and analysis provided to allow replication by others?

No

Reviewer Expertise:

Health Economic, health insurance, and disesase prevention

I confirm that I have read this submission and believe that I have an appropriate level of expertise to confirm that it is of an acceptable scientific standard, however I have significant reservations, as outlined above.

# Response

**Published:** 2025-01-07

**Authors:** rajesh kamath

**Affiliations:** Prasanna School of Public Heal, Manipal Academy of Higher Education, Manipal, Karnataka, India

**Introduction:**

**1. There is insufficient emphasis on the importance of selecting specific types of operations to be analyzed or researched:** 

**Response: **The selection of surgeries—Cesarean Section (C-Section), Laparoscopic Hysterectomy, Laparoscopic Cystectomy, Laparoscopic Myomectomy, and Laparoscopic Assisted Vaginal Hysterectomy (LAVH)—was based on their high prevalence within the hospital’s obstetrics and gynecology department. These five surgeries were the top five most commonly performed surgeries during the study period. These surgeries were selected as they provide a comprehensive representation of the department’s surgical workload, allowing a focused analysis of OOPE trends among the most commonly encountered obstetrics and gynecology surgeries. And the same was mentioned in the last paragraph of the discussion part “C-Section, laparoscopic hysterectomy, laparoscopic cystectomy, laparoscopic myomectomy and LAVH were the top five most performed obstetrics and gynecology surgeries in the tertiary care teaching hospital between January 2023 and July 2023 and scrutinizing spending patterns associated with these five surgeries can provide precise insights for refining policies to enhance the accessibility of obstetrics and gynaecology patientsand to mitigate the financial burden.”

**2. The study does not address the local context in Karnataka, particularly the geographical or socio-economic differences that may influence OOPE (Out-of-Pocket Expenditure).**

**Response:**The study primarily aimed to analyze OOPE patterns for five most common obstetrics and gynecology surgeries within the framework of a tertiary care teaching hospital in Karnataka. While the local context is implicitly addressed through the study setting, which serves as a referral center for a diverse population spanning rural and urban regions of Karnataka, the study did not specifically delve into variations based on socio-economic or geographical factors. By focusing on the hospital's patient population, the study aimed to offer insights that are actionable for policymakers, while laying the groundwork for future research to examine regional variations in OOPE more comprehensively.

**3.Not yet linking the impact of OOPE with catastrophic conditions that can have an impact on poverty**

**Response:** We have made changes and addressed this in the second last paragraph of the introduction part. The revision explains how high OOPE can lead to catastrophic health expenditure, forcing households into poverty, and the role of government schemes like AB-PMJAY in alleviating this impact.

**Method:**

**1.There is no clear definition of OOPE in the method and other variables that significant influencing the OOPE incident**

**Response:** We have addressed the definition of Out-of-Pocket Expenditure (OOPE) in the Statistical Method ****section of the Method part of the study. 

**2.Econometric and multivariate analysis**

**Response:** The study was designed as a retrospective descriptive analysis using descriptive statistics such as means, medians, and the Shapiro-Wilk test for normality. While econometric and multivariate approaches could provide additional insights, they were beyond the scope of this study, given the available retrospective data and objectives.

 Nevertheless, the descriptive approach provided sufficient detail to highlight stark disparities in OOPE, such as the zero OOPE for AB-PMJAY beneficiaries compared to the median ₹33,257 ($406) incurred by uninsured patients for C-Sections.

**Results:**

**1. The graphs should have more in-depth interpretation.**

**Response:** Graphs were used to visually present data trends across surgeries and patient categories, providing readers with a clear understanding of OOPE variations. For example, Figure 18 demonstrates the median OOPE for uninsured patients across surgeries, which ranged from ₹33,257 ($406) for C-Sections to ₹57,053 ($696) for Laparoscopic Hysterectomies. The accompanying text explains these trends, highlighting the effectiveness of AB-PMJAY and the burden on uninsured patients. Expanding interpretations further could lead to redundancy, as the key observations are already summarized in the discussion section.

**2.Too many graph, maybe some of them can be combined or adjusted**

**Response:** Each graph serves a distinct purpose by illustrating specific aspects of the results, such as OOPE variations by surgery type, patient category, and type of insurance. For example, Figures 3 to 6 detail the minimum, maximum, mean, and median OOPE for different surgeries, while Figure 17 compares the average OOPE across patient categories. Combining these graphs risks oversimplifying the findings, potentially obscuring important details.

**3.There is insufficient emphasis on the social impact, particularly on uninsured patients.**

**Response:** The social implications of OOPE are indirectly addressed through the analysis of financial burdens across patient categories. For instance, uninsured patients faced significantly higher OOPE, with an average of ₹53,151 ($648.18) for LAVH, compared to ₹7,229 ($88.17) for private health insurance patients. This highlights the inequities in financial risk protection and the need for expanded insurance coverage. While the primary focus was on quantifying OOPE, the findings inherently underscore its social impact.

**Discussion:**

**1.The discussion is overly focused on the data presented without comparing it to previous studies.**

**Response:** As mentioned in the very first sentence of the discussion, "There is little existing peer-reviewed literature on OOPE among obstetrics and gynaecology patients." This highlights the limited availability of prior research specifically focused on this subject area, making it challenging to provide a direct comparison. The study, therefore, serves as an important contribution to filling this knowledge gap by offering new insights into OOPE incurred by patients undergoing common obstetrics and gynaecology surgical procedures. Future research can build upon this study to facilitate more robust comparative analyses as additional data becomes available.

 2. **The discussion merely reiterates the results without analyzing the reasons behind differences in OOPE among groups.**

**Response:** We have addressed this in the third paragraph of the discussion. We have provided a detailed explanation for the variations in OOPE across the groups.

**3.It does not address the impact of OOPE on patients.**

**Response:** The discussion does address the impact of OOPE on patients, particularly in terms of how high OOPE can create financial strain, limit access to timely healthcare, and affect health outcomes. For instance, the discussion highlights that uninsured patients faced substantial OOPE, ranging from Rs. 33,257 for a C-section to Rs. 57,053 for laparoscopic cystectomy, which could lead to financial hardship and adverse health outcomes. Additionally, it points out that OOPE remains a critical barrier to accessing quality obstetric and gynecological care, especially for disadvantaged socioeconomic groups, and that reducing OOPE through schemes like AB-PMJAY enhances financial protection, thus facilitating access to necessary surgeries and potentially mitigating maternal mortality. This analysis underscores the importance of reducing OOPE to improve access to healthcare and reduce health disparities among different patient groups.

**4.There is no detailed explanation of whether other factors, such as geographical location, educational level, or type of healthcare facility, influenced the results.**

**Response:** The study does not delve into factors such as geographical location, educational level, or type of healthcare facility, as it was a retrospective study conducted within a single tertiary care teaching hospital in Karnataka. The study's focus was primarily on comparing OOPE across different insurance schemes (AB-PMJAY, private insurance, and uninsured patients), and the sample was limited to patients within this specific setting. However, acknowledging that these factors could potentially influence OOPE, future studies could explore their role across a more diverse range of healthcare settings and regions to provide a broader understanding of how these variables affect out-of-pocket expenses.

**5.There is no clear explanation of how these findings can influence policymakers, insurance providers, or hospitals.**

**Response:** In the discussion, the study emphasizes the role of government-funded health insurance initiatives like AB-PMJAY in mitigating OOPE and promoting greater healthcare access, especially for vulnerable population. It also highlights the limitations of private insurance in fully covering the costs of surgeries for obstetrics and gynecology patients. These findings suggest that expanding government insurance schemes and refining private health insurance coverage could reduce OOPE and improve healthcare equity. Moreover, the study advocates for increasing participation in government health schemes to alleviate financial barriers, which can guide policymakers, insurance providers, and hospitals in improving healthcare accessibility and affordability.

**Conclusion:**

**1.The study does not provide a conclusion summarizing the research findings.**

**Response:** The discussion in the article provides a clear summary of the research findings, particularly in the comparison of OOPE across AB-PMJAY, private health insurance, and uninsured patients. It highlights how AB-PMJAY significantly reduced OOPE, while private health insurance did not fully alleviate the financial burden for patients. This summary of key findings can be considered a conclusion, reflecting the effectiveness of government health insurance and the need for further improvements in private insurance (as discussed in the sentences: "The study demonstrates the effectiveness of AB-PMJAY in reducing OOPE...").

**2.There are no specific recommendations for health insurance companies or hospitals to prevent OOPE in patients in the future.**

**Response:** ​​​​​​​While the study does not explicitly offer recommendations, it indirectly suggests areas for improvement in insurance coverage and healthcare policies. For instance, the discussion mentions that expanding government-funded insurance schemes like AB-PMJAY is crucial to mitigating financial barriers for patients, particularly for obstetrics and gynaecology services. Additionally, it points out that private insurance may not sufficiently cover surgical costs, which can serve as a recommendation for policy adjustments to better support patients ("Expanding government-funded insurance initiatives like AB-PMJAY is critical to mitigating inequities...").

# Reviewer report: Reviewer response for version 1

**DOI:** 10.5256/f1000research.172621.r348062

**Published:** 2024-12-24

**Authors:** Neha Ahire

**Affiliations:** 1 Symbiosis Institute of Health Sciences Symbiosis International (Deemed University), Pune, India

**Work presentation and Literature review**

**Timely and Relevant Topic**: The study addresses an extremely pertinent issue in the Indian healthcare system— **Out-of-Pocket Expenditure (OOPE)**. Given that a significant portion of the Indian population remains uninsured or underinsured, this issue directly impacts access to healthcare services. The study is timely, especially in light of the increasing adoption of schemes like **AB-PMJAY**, which aims to provide healthcare for the economically disadvantaged.

- **Focus on Obstetrics and Gynecology**: By focusing on **Cesarean Section (C-Section)** and other major gynecological surgeries, the study highlights in on a critical area of healthcare, as maternal and reproductive health is a major concern in India.

Methodology - The methodology is described in sufficient detail to allow replication, including data collection and analysis methods. The use of the Shapiro-Wilk test for normality adds robustness, but supplementary explanations on why certain data distribution assumptions were made would enhance clarity.

 Analysis : The statistical analyses are generally appropriate, but the results could benefit from a deeper exploration of disparities among private health insurance providers. 

 Data Source  Source data is openly available, which supports transparency and reproducibility. The inclusion of anonymized data sets and validated proformas is commendable.

 The conclusions are well-supported by the presented data and effectively highlight the role of AB-PMJAY in reducing OOPE. However, further discussion of policy implications and potential strategies to enhance the effectiveness of private insurance would enrich the conclusion.

**Recommendations: **This study offers significant contributions to understanding OOPE in obstetrics and gynaecology but could benefit from the following minor revisions:

 1. Sample size - The term "905 maternal patients" to be replaced by appropriate term. 

 2.  Expand the discussion on the variation in OOPE among different private insurers.

 Include a justification for the selection of specific surgeries and their broader relevance to maternal healthcare.

 3. The study is conducted in a **single tertiary care hospital** in Karnataka. While this provides in-depth insights into the OOPE experienced by patients in this specific setting, the results may not be generalizable to other parts of India. This should be added as limitation or generalization of the findings to be supported by the latest index.

Is the work clearly and accurately presented and does it cite the current literature?

Yes

If applicable, is the statistical analysis and its interpretation appropriate?

Yes

Are all the source data underlying the results available to ensure full reproducibility?

Yes

Is the study design appropriate and is the work technically sound?

Yes

Are the conclusions drawn adequately supported by the results?

Yes

Are sufficient details of methods and analysis provided to allow replication by others?

Yes

Reviewer Expertise:

Health Insurance , Universal Health Coverage

I confirm that I have read this submission and believe that I have an appropriate level of expertise to confirm that it is of an acceptable scientific standard.
